# Effect of population structure and stabilizing selection on quantitative genetic variation

**DOI:** 10.1093/genetics/iyag185

**Published:** 2026-07-21

**Authors:** Juan Li, Joachim Hermisson, Himani Sachdeva

**Affiliations:** Faculty of Mathematics, University of Vienna, Oskar-Morgenstern-Platz 1, Vienna, 1090, Austria; Faculty of Mathematics, University of Vienna, Oskar-Morgenstern-Platz 1, Vienna, 1090, Austria; Max Perutz Labs, University of Vienna, Dr. Bohrgasse 9, Vienna, 1030, Austria; Faculty of Mathematics, University of Vienna, Oskar-Morgenstern-Platz 1, Vienna, 1090, Austria; Department of Evolutionary Biology, University of Vienna, Djerassiplatz 1, Vienna, 1030, Austria

**Keywords:** polygenic traits, stabilizing selection, population structure, genetic variance, linkage disequilibrium, *F*_*ST*_–*Q*_*ST*_ comparison

## Abstract

We study one of the simplest scenarios of polygenic selection that can be imagined: a subdivided population of diploid individuals expressing an additive trait under spatially homogeneous stabilizing selection. We are interested in the amounts of variation that can be maintained at mutation-selection-migration-drift equilibrium, at individual loci and at the level of the trait, within and among subpopulations. We derive analytical approximations for variance components and summary statistics such as $F_{ST}$ and $Q_{ST}$ under the assumptions of the infinite-island model and compare these with individual-based simulations. We find that: (i) There is a critical migration threshold (which depends on effect sizes of trait loci) below which population structure strongly inflates genic variance in the subdivided population to levels well above those in a panmictic population. Variation within each subpopulation is maximized close to the critical migration rate. (ii) The genetic basis of trait variation across subpopulations is most similar close to this migration threshold and (counter-intuitively) becomes less similar for higher migration rates. This has consequences for the “portability” of Genome-Wide Association Studies between subpopulations, i.e. the extent to which loci with large contributions to variance in one subpopulation explain variance in other subpopulations. (iii) An analytical mean-field approach based on the single-locus diffusion approximation, together with effective migration and selection parameters (to account for associations between loci), very accurately predicts various quantities.

## Introduction

Large, outbreeding populations typically harbor high levels of molecular and phenotypic variation. However, we still do not fully understand how such variation emerges from the interplay between different evolutionary processes (Ch. 28, Walsh and Lynch 2018). Early work focused on whether high trait variation might reflect a balance between mutation and stabilizing selection toward an intermediate trait optimum (Lande 1975; Turelli 1984; Bürger et al. 1989). More recent work suggests that a model of stabilizing selection acting on multiple traits is broadly consistent with the findings of Genome-Wide Association Studies (GWAS) for a suite of quantitative traits in humans (Simons et al. 2018, 2025).

This still leaves questions as to how much other factors, such as balancing or fluctuating selection and spatial population structure, contribute to the maintenance of variation, and whether observed patterns of variation might be consistent with several alternative evolutionary histories and scenarios (Barton and Keightley 2002; Sella and Barton 2019). Theoretical models show that selection that varies across time (Bürger and Gimelfarb 2002; Bertram and Shafiei 2026) or space (Slatkin 1978; Barton 1999; Tufto 2000) can significantly inflate genetic variation under certain conditions. However, most of this work focuses on extreme cases, e.g. strongly selected loci (that evolve deterministically) vs. the infinitesimal model. This makes it difficult to generalize their conclusions to quantitative trait variation which may have a disproportionate contribution from loci of small to intermediate effect (Simons et al. 2018).

One general mechanism by which spatial population structure can increase genetic variability is if different subpopulations occupy different “adaptive peaks,” i.e. harbor distinct genetic combinations that are approximately equally fit (Wright 1935). Alternative fitness peaks might arise due to spatially heterogeneous selection, e.g. if different trait values are favored across different geographic regions or ecological niches. However, they can also arise under spatially uniform selection, provided that the genetic architecture of selected traits is sufficiently “redundant,” i.e. if many genetic combinations correspond to approximately the same phenotype (Goldstein and Holsinger 1992; Phillips 1996; Lythgoe 1997; Barghi et al. 2020).

There has been much interest recently in how such genetic redundancy in adaptive traits could lead to (dis-)similar genomic signatures of adaptation in natural or experimental replicates (MacPherson and Nuismer 2017; Yeaman et al. 2018; Barghi et al. 2019; Höllinger et al. 2023). However, all of this work assumes that replicate populations evolve independently. By contrast, most natural populations consist of sub-populations connected by historical or ongoing gene flow, which may cause the genetic basis of trait variation to become more similar across subpopulations, despite redundancy. This leads us to ask: do the same or different loci contribute to variation across different subpopulations? And how does this depend on selection and the genetic architecture of traits on the one hand, and dispersal and demography on the other? Put another way, to what extent does selection on polygenic traits perturb patterns of allele sharing at trait loci in a structured population, causing these to stand out from the neutral genomic background?

These questions are relevant to various efforts to predict phenotypes and make inferences about selection from genomic data while accounting for population structure. For instance, how well genomic prediction based on GWAS generalizes across populations depends crucially on the extent to which the same genetic variants underlie phenotypic variation across populations with different ancestry (Duncan et al. 2019; Wang et al. 2020; Yair and Coop 2022). Similarly, our ability to distinguish signatures of local vs. global adaptation in genomic data also depends on whether the same or different alleles underlie (either kind of) adaptation in different sub-populations (Ralph and Coop 2015; Booker et al. 2021). However, even low levels of gene flow between populations can qualitatively alter theoretical expectations, which makes it crucial to model the combined effects of selection and population structure on trait variation.

As a step in this direction, we analyze the simplest possible scenario of a polygenic trait subject to spatially uniform stabilizing selection across a large number of “islands” or sub-populations connected via migration. We explore the effects of selection and structure on diversity at individual trait loci as well on the total trait variance, which also depends on statistical associations (or linkage disequilibria; LD) between trait loci. The goal is to describe and predict patterns of variation at different “scales”: globally (across the full population) vs locally (within sub-populations), and at the locus vs. the trait level.

Previous work on the effect of (uniform) selection and spatial structure on quantitative genetic (QG) variation is largely simulation-based (Goldstein and Holsinger 1992; Latta 1998; Le Corre and Kremer 2003, 2012; McDonald and Yeaman 2018) or, otherwise, derives analytical results by assuming strong selection and deterministic evolution (Phillips 1996; Lythgoe 1997). This makes it difficult to link theory to patterns in genomic variation, which are significantly affected by genetic drift. Here, we address this gap by developing detailed analytical theory that accounts for the combined effects of mutation, stabilizing selection, drift, and population structure on trait variants, thus allowing for a more comprehensive understanding. Our analysis relies on the fact that stabilizing selection on a polygenic trait primarily generates selection against variance (at least if the trait mean is close to the trait optimum). This generates selection against the minor allele at any trait locus, resulting in the same evolutionary dynamics as at a locus with underdominance(Wright 1935; Robertson 1956; Bulmer 1972). Thus, as a first approximation, we can treat the polygenic trait as being determined by a set of *independently* evolving underdominant loci. This allows us to use single-locus theory, in particular, the diffusion approximation to study the stochastic evolution of allele frequencies at trait loci (Wright 1937; Bulmer 1972; Barton and Rouhani 1993). We then build upon this to account for the effects of LD between trait loci using effective parameters. As we demonstrate below, this approach yields accurate predictions for gene-level as well as trait-level variation across most of parameter space, and also extends to traits influenced by a broad distribution of effect sizes.

A natural reference point for our analysis is the strong migration limit of the model, in which the subdivided population resembles a very large, panmictic population. The total genetic variance at mutation-selection equilibrium in such a population depends only on the total mutation rate for the trait, and the strength of stabilizing selection (Latter 1960; Bulmer 1972), and is independent of the effect size of trait loci, at least in the House-of-Cards mutation regime (Turelli 1984). How might this change in scenarios with limited migration between subpopulations? In particular, do loci with certain effect sizes contribute disproportionately to variability within or between subpopulations, making them more likely to drive adaptive response and/or be identified in genomic data?

## Model and methods

### Model

We consider a subdivided population consisting of *D* demes (islands), each with *N* diploid individuals. For theoretical analyzes, we will take the limit D→∞, which corresponds to the infinite-island model, introduced by Wright (1931). Demes are connected via migration: in each generation, a migrant pool is formed by drawing individuals from across the whole population, with migration probability *m* per individual. Individuals from this pool are then uniformly redistributed back across all demes of the population. Thus, *Nm* represents the average number of migrants exchanged between a deme and the rest of the population.

Each individual expresses an additive trait influenced by *L* unlinked (freely recombining), bi-allelic loci. Alternative alleles at any locus are denoted by X=0,1; the genotype of an individual *j* at locus *i* is represented as $\left(X_{ij}{\tilde{X}}_{ij}\right)$, where $X_{ij}$ and ${\tilde{X}}_{ij}$ denote maternally and paternally inherited alleles. Thus, there are 3 possible genotypes: (00), (01) (which is equivalent to (10)), and (11) at locus *i*, with contributions to trait value given by $-\alpha_{i}$, 0, and $\alpha_{i}$ respectively. Hereafter, we refer to $\alpha_{i}$ as the effect size at locus *i*. The trait value $z_{j}$ of an individual *j* is given by:


(1) $$z_{j}=\sum_{i=1}^{L}\alpha_{i}(X_{ij}+{\tilde{X}}_{ij}-1)$$

For simplicity, we assume that the trait has no environmental component. However, this assumption is not crucial, since a nonzero environmental variance only rescales the effective strength of stabilizing selection on the trait (see, e.g. Bürger et al. 1989). Polymorphism is maintained by mutation, which occurs at rate *μ* per allele per individual per generation. Thus, the expected number of trait-affecting mutations per diploid individual per generation, is 2μL.

The trait is assumed to be under stabilizing selection, which disfavors individuals with trait values that deviate from the optimum. The fitness of an individual with trait value *z* is:


(2) $$W(z)=e^{-\frac{(z-z_{\mathrm{opt}}{)}^{2}}{2V_{s}}}$$

The trait optimum, $z_{\mathrm{opt}}$, and the width of the fitness function, $\sqrt{V_{s}}$, is the *same* across all demes, resulting in spatially *uniform* selection across the entire population.

Finally, we assume that individuals mate randomly within demes based on the Wright–Fisher model. Thus, the next generation is formed by sampling 2N parents (with replacement) within each deme, with sampling weights proportional to the relative fitness of individuals in that deme. Each of these 2N parents produce haploid gametes via free recombination between trait loci. Gametes are then paired randomly to form *N* diploid offspring, which replace the parents.

All 3 processes—stabilizing selection, migration, and drift—can generate LD between trait loci within demes, thus causing them to evolve in a nonindependent manner. If recombination is much faster than other evolutionary processes, such LD is weak, and can be neglected to a first approximation. This is the basis of our linkage equilibrium (LE) approximation, which accounts for the effects of migration, mutation, genetic drift, and direct selection (arising from its own effect) at each trait locus, but not those of LD between loci. However, LD cannot always be ignored, particularly with intermediate levels of population structure, and if traits have a large total mutation rate 2μL. Therefore, we extend our approximations to account for the effects of LD using appropriately defined effective parameters, which leads to more accurate predictions for the variance contributions of trait loci. Finally, we develop predictions for the variance of trait values, and how it is partitioned within and between demes.

### Allele frequency distributions at trait loci under LE

Following Bulmer (1972), we describe the evolution of allele frequencies at trait loci in continuous time. To approximate the dynamics of the discrete generation model via continuous time equations, we will assume that the distribution $\rho_{j}(z)$ of trait values within any (say, the jth) deme is normal with mean ${\bar{z}}_{j}$ and variance $V_{W,j}$, i.e.


(3) $$\rho_{j}(z)=\frac{1}{\sqrt{2\pi V_{W,j}}}e^{-\frac{(z-{\bar{z}}_{j}{)}^{2}}{2V_{W,j}}}$$

Here, $V_{W,j}$ is the *within*-deme phenotypic variance, which is equal to the genetic variance (since trait values are assumed to have no environmental component).

The mean fitness in the deme is then given by:


(4) $$\begin{array}{rl} {\bar{W}}_{j}=\int_{-\infty }^{\infty }W(z)\rho_{j}(z)\;dz & =\exp\;\left[-\frac{({\bar{z}}_{j}-z_{\mathrm{opt}}{)}^{2}}{2V_{s}\left(1+\frac{V_{W,j}}{V_{s}}\right)}-\frac{1}{2}\log\;\left(1+\frac{V_{W,j}}{V_{s}}\right)\right]\\ & \approx \exp\;\left[-\frac{({\bar{z}}_{j}-z_{\mathrm{opt}}{)}^{2}}{2V_{s}}-\frac{V_{W,j}}{2V_{s}}\right]\;\text{for}\;V_{w,j}\ll V_{s} \end{array}$$

Thus, for $V_{W}\ll V_{s}$, the reduction in (log) mean fitness has 2 distinct components—one depending on $({\bar{z}}_{j}-z_{\text{opt}})/\sqrt{V_{s}}$, the deviation of the trait mean from the trait optimum, and the other on $\sqrt{V_{W,j}/V_{s}}$, the standard deviation of trait values, both expressed relative to the width of the stabilizing selection function.

The trait mean can be expressed in terms of the allele frequencies as ${\bar{z}}_{j}=\sum_{i=1}^{L}\alpha_{i}(p_{i,j}-q_{i,j})$, where $p_{i,j}$ and $q_{i,j}=1-p_{i,j}$ denote, respectively, the frequencies of the trait-increasing and trait-decreasing alleles at locus *i* in deme *j*. The genetic variance, $V_{W}$, has 2 components: a genic component, $V_{W}^{\left(gen\right)}$, and a component due to LD, $V_{W}^{\left(LD\right)}$. If recombination between trait loci is much stronger than selection and gene flow, individual demes are approximately in LE, so that: $V_{W}^{\left(LD\right)}\approx 0$ and $V_{W,j}\approx V_{W,j}^{\left(gen\right)}=2\sum_{i=1}^{L}\alpha_{i}^{2}p_{i,j}q_{i,j}$. Then, the selection gradient at trait locus *i* in deme *j* is simply:


(5) $$\frac{\partial \log\;({\bar{W}}_{j})}{\partial p_{i,j}}=-2\frac{\alpha_{i}}{V_{s}}({\bar{z}}_{j}-z_{\mathrm{opt}})-\frac{\alpha_{i}^{2}}{V_{s}}(q_{i,j}-p_{i,j})$$

Note that the same expression for the selection gradient can be obtained without assuming a normal distribution of trait values, if we start with a model of continuous time evolution and assume LE (Höllinger et al. 2023).

The trait mean within any deme rapidly approaches the trait optimum, at least if there is no mutational bias and the optimum is close to the center of the phenotypic range, i.e. $z_{\mathrm{opt}}\approx 0$ (see also Bulmer 1971). Thus, in a population at equilibrium, the selection gradient at any locus is approximately $-\frac{\alpha_{i}^{2}}{V_{s}}(q_{i,j}-p_{i,j})$. Furthermore, if mutation, migration and selection per locus are sufficiently weak, i.e. if $\mu ,m,\alpha_{i}^{2}/V_{s}\ll 1$, then we can describe the deterministic evolution of allele frequencies (neglecting drift) in continuous time as follows:


(6) $$\frac{\text{d}p_{i,j}}{\text{dt}}=-\frac{\alpha_{i}^{2}}{2V_{s}}p_{i,j}q_{i,j}(q_{i,j}-p_{i,j})+\mu (q_{i,j}-p_{i,j})+m({\bar{\;p}}_{i}-p_{i,j})$$

The first term describes the effects of selection on allele frequencies, and has exactly the same form as for a single underdominant locus with heterozygote disadvantage $s_{i}=\frac{\alpha_{i}^{2}}{2V_{s}}$. The second term describes the effects of mutation, which tends to drive allele frequencies toward 1/2 (in the case of symmetric mutation). The third term describes the effects of migration that pulls allele frequencies toward ${\bar{\;p}}_{i}$, the mean allele frequency (at locus *i*) in the migrant pool, which is equal to the mean allele frequency across the entire population under the infinite-island assumption.

Thus, allele frequencies at any locus underlying a trait under stabilizing selection in a subdivided population follow relatively simple dynamics, which are identical to those of a single underdominant locus. As discussed above, this holds if: first, genetic variance within demes is sufficiently small, i.e. $V_{W}/V_{S}\ll 1$; second, LD between trait loci is negligible; third, the trait mean in each deme is close to the trait optimum, i.e. ${\bar{z}}_{j}\approx z_{\mathrm{opt}}$. In subsequent sections, we will relax the first and second assumptions.

Equation (6) describes the deterministic dynamics of allele frequencies in a large population under LE. However, genetic drift generates additional random fluctuations, with the variance of random allele frequency change per generation due to drift being equal to: $\text{Var}[\Delta p_{i,j}]=p_{i,j}q_{i,j}/(2N)$ within any deme. We can describe the combined effects of drift, migration, mutation, and selection on allele frequencies using the diffusion approximation (Kimura 1964). In particular, under the infinite-island model (i.e. in the limit D→∞), allele frequencies $\left\{p_{i,j}\right\}$ across different demes j=1,…D (at say, locus *i*) are mutually *independent* random variables that depend only on the mean allele frequency ${\bar{\;p}}_{i}$ in the migrant pool. From equation (6), it follows that the equilibrium distribution of $p_{i,j}$ in any deme *j*, conditioned on ${\bar{\;p}}_{i}$ is:


(7) $$\Phi (p_{i,j}\;|\;{\bar{\;p}}_{i})=C\;e^{-4Ns_{i}p_{i,j}q_{i,j}}p_{i,j}^{4N\mu +4Nm{\bar{p}}_{i}-1}q_{i,j}^{4N\mu +4Nm{\bar{q}}_{i}-1}$$

where *C* is the normalization constant (see also Wright 1937). In the limit D→∞, the variance of ${\bar{p}}_{i}$ approaches zero. Thus, the allele frequency in the migrant pool, ${\bar{\;p}}_{i}$, must be *deterministically* equal to the expected allele frequency within demes. In other words, we have:


(8) $${\bar{\;p}}_{i}=E[p\;|\;{\bar{p}}_{i}]=\int_{0}^{1}p\;\Phi (p\;|\;{\bar{p}}_{i})\;\text{d}p$$

This can be solved numerically (see also Barton and Rouhani 1993), thus allowing us to calculate various other quantities by integrating over equation (7). We refer to the predictions obtained in this way as LE predictions. In particular, one can express $V_{W}^{\left(gen\right)}$ and $V_{T}^{\left(gen\right)}$, the expected *genic* variance within demes and across the total population as:


(9) $$V_{W}^{\left(gen\right)}=2\sum_{i=1}^{L}\alpha_{i}^{2}E[p_{i}q_{i}],\;V_{T}^{\left(gen\right)}=2\sum_{i=1}^{L}\alpha_{i}^{2}{\bar{p}}_{i}{\bar{q}}_{i}$$

Here, ${\bar{\;p}}_{i}$ and $E[p_{i}q_{i}]$ can be obtained from equation (7) via numerical integration. In addition, we derive simple analytical expressions for these quantities in the weak selection (4Nμ<Ns≲1) and strong selection (Nμ≪1≲Ns) limits (Supplementary Sec. SI.a).

In the following, we will report trait variances in units of $V_{*}$, the genic variance in an infinitely large panmictic population at mutation-selection equilibrium. Setting m=0 in equation (6), it follows that at mutation-selection equilibrium, we have $p_{i}q_{i}=\mu /s_{i}$, provided $4\mu <s_{i}$. This gives: $V_{*}=4\mu LV_{s}$, which is proportional to the total mutation rate for the trait and independent of effect sizes (Turelli 1984).

Thus, the *scaled* genic variances can be expressed as:


(10) $$\frac{V_{W}^{\left(gen\right)}}{V_{*}}=\frac{2\sum_{i=1}^{L}\alpha_{i}^{2}E[p_{i}q_{i}]}{4\mu LV_{s}}=\frac{1}{L}\sum_{i=1}^{L}\frac{Ns_{i}}{N\mu }E[p_{i}q_{i}],\;\frac{V_{T}^{\left(gen\right)}}{V_{*}}=\frac{1}{L}\sum_{i=1}^{L}\frac{Ns_{i}}{N\mu }{\bar{p}}_{i}{\bar{q}}_{i}$$

Since ${\bar{\;p}}_{i}$ and $E[p_{i}q_{i}]$ only depend on the *Ns*, Nμ and *Nm* (equation (7)), this allows us to express our results purely in terms of scaled (dimensionless) parameters.

#### Distribution of variance contributions of trait loci

In addition to predicting the expected genic variance, the equilibrium allele frequency distribution $\Phi (p\;|\;\bar{p})$ (equation (7)) can also be used to predict the *distribution* of variance contributions of individual loci within demes. As before, we scale the variance contribution *v* of any locus by the deterministic expectation $v_{*}$ (which is $4\mu V_{s}$ for a single locus), and denote the scaled variance by $\tilde{v}=v/v_{*}$. A locus with effect size *α* (or selection coefficient $s=\frac{\alpha^{2}}{2V_{s}}$) and allele frequency *p* within a deme has a (scaled) variance contribution $\tilde{v}=\frac{2\alpha^{2}pq}{4\mu V_{s}}=\frac{Ns}{N\mu }pq$. Transforming variables from *p* to $\tilde{v}$ in equation (7) gives the following expression for the the probability density $P(\tilde{v}\;|\;s)$ of variance contributions:


(11) $$P(\tilde{v}\;|\;s)=\frac{Ns}{N\mu }\frac{\Phi (p_{+}\;|\;\bar{p})+\Phi (p_{-}\;|\;\bar{p})}{\sqrt{1-4\frac{N\mu }{Ns}\tilde{v}}}\;\text{where}\;p_{\pm }=\frac{1\pm \sqrt{1-4\frac{N\mu }{Ns}\tilde{v}}}{2}$$

The cumulative probability that a trait locus with selective effect *s* contributes an amount *exceeding y* (in units of $v_{*}$) to the genic variance within a deme is then:


$$F(y\;|\;s)=P(\tilde{v}>y\;|\;s)=\int_{y}^{{\tilde{v}}_{\max}}P(\tilde{v}\;|\;s)\;\text{d}\tilde{v}$$

where ${\tilde{v}}_{\max}=\frac{Ns}{4N\mu }$ is the maximum possible value of $\tilde{v}$ (corresponding to p=1/2).

Similarly, we can define $P_{2}({\tilde{v}}_{1},{\tilde{v}}_{2}\;|\;s)$ as the joint probability density of variance contributions of a locus with selective effect *s* in 2 demes (arbitrarily labeled “1” and “2”). The corresponding joint cumulative distribution is: $F_{2}(y_{1},y_{2}\;|\;s)=P({\tilde{v}}_{1}>y_{1},{\tilde{v}}_{2}>y_{2}\;|\;s)$. Since allele frequencies in different demes are independent under the infinite-island model (conditional on the mean $\bar{p}$), we have: $P_{2}({\tilde{v}}_{1},{\tilde{v}}_{2}\;|\;s)=P({\tilde{v}}_{1}\;|\;s)P({\tilde{v}}_{2}\;|\;s)$ and $F_{2}(y_{1},y_{2}\;|\;s)=F(y_{1}\;|\;s)F(y_{2}\;|\;s)$.

In general, trait loci will have unequal effects, leading to a distribution of selective effects, which we denote by ψ[s]. The cumulative distributions F(y) and $F_{2}(y_{1},y_{2})$ across all trait loci can then be obtained by integrating over ψ[s]:


(12) $$F(y)=\int_{s}F(y\;|\;s)\psi [s]\;\text{d}s\;F_{2}(y_{1},y_{2})=\int_{s}F(y_{1}\;|\;s)F(y_{2}\;|\;s)\psi [s]\;\text{d}s$$

### Accounting for LD using effective parameters

So far, we have neglected the effects of LD between trait loci within demes. However, both stabilizing selection and migration build up LD, leading to nonindependent evolution of trait loci. In this section, we will discuss each of these two sources of LD, and describe how the effects of LD on a given locus can be encapsulated via a suitably defined effective selection coefficient and effective migration rate. These effective rates depend on allele frequencies at all other trait loci, allowing us to obtain a mean field (i.e. self-consistent) solution for all the allele frequencies jointly. This approach of representing the effects of LD using effective parameters is very similar in spirit to the quasi linkage equilibrium approximation and applies as long as the dynamics of LD are much faster than the dynamics of allele frequencies (Kimura 1965). This, in turn, requires recombination to be much faster than all other processes that influence single-locus dynamics, which is the case in a model with unlinked loci.

First, consider the effects of stabilizing selection, which generates negative LD between trait-increasing (or, equivalently, trait-decreasing) alleles within a deme. This causes the genetic variance (i.e. the variance of trait values) to be less than the genic variance (Bulmer 1971). To quantify this reduction, we assume (as before) that *L* is large enough that the distribution ρ(z) of trait values within any deme is approximately normal with mean $\bar{z}$ and variance $V_{W}$. Stabilizing selection changes the distribution of trait values to: $\rho^{\prime }(z)=\rho (z)(W(z)/\bar{W})$, where $\bar{W}$ is given by equation (4). This shifts the trait mean toward the trait optimum and decreases the trait variance, so that after selection, we have:


(13) $${\bar{z}}^{\prime }=\frac{V_{W}\;z_{\mathrm{opt}}+V_{s}\;\bar{z}}{V_{W}+V_{s}}\;V_{W}^{\prime }=\frac{V_{W}\;V_{s}}{V_{W}+V_{s}}$$

Random mating within demes leaves the trait mean unchanged (${\bar{z}}^{\prime \prime }={\bar{z}}^{\prime }$), but changes the trait variance to: $V_{W}^{\prime \prime }=(V_{W}^{\prime }/2)+(V_{W}^{\left(gen\right)}/2)$. Here, $V_{W}^{\left(gen\right)}/2$ is the segregation variance (also known as the within-family variance) released by recombination between parental haplotypes. For a randomly mating population, the segregation variance is equal to half of the genic variance. For a population at equilibrium, the trait value distributions do not change from one generation to the next. Thus, we have: ${\bar{z}}^{\prime \prime }=\bar{z}$ and $V_{W}^{\prime \prime }=V_{W}$, which gives:


(14) $$\begin{array}{rl} \frac{V_{W}}{V_{W}^{\left(gen\right)}} & =\frac{1}{4}\left(1-\frac{V_{s}}{V_{W}^{\left(gen\right)}}+\sqrt{1+6\frac{V_{s}}{V_{W}^{\left(gen\right)}}+{\left(\frac{V_{s}}{V_{W}^{\left(gen\right)}}\right)}^{2}}\right)\\ & \approx 1-\frac{V_{W}^{\left(gen\right)}}{V_{s}}+O\left[{\left(V_{W}^{\left(gen\right)}/V_{s}\right)}^{2}\right] \end{array}$$

which is equivalent to equations 18 and 22 of Bulmer (1971). Thus, for $V_{W}^{\left(gen\right)}/V_{s}\ll 1$, the genetic variance within a deme is reduced relative to genic variance by an amount $\approx V_{W}^{\left(gen\right)}/V_{s}$, which is twice the genetic load per deme.

In addition to reducing genetic variance, negative LD between trait loci also generates indirect selection at each locus by causing the trait-increasing allele at any locus to become associated with alleles of opposite effect at other loci. This slightly reduces the average effect of the allele (e.g. as estimated by GWAS), which slightly relaxes heterozygote disadvantage and increases heterozygosity at each locus, thus causing the genic variance to be *higher* than the LE prediction. Further, in parameter regimes where negative LD is significant (i.e. when $V_{W}^{\left(gen\right)}/V_{s}$ is nonnegligible), the approximation ${\bar{W}}_{j}\approx e^{-\frac{1}{2V_{s}}[({\bar{z}}_{j}-z_{\mathrm{opt}}{)}^{2}+V_{W,j}]}$ (second line of equation (4)) also becomes less accurate. Instead, we must use the full expression in equation (4) to calculate the (direct) selection gradient any locus. Following Veller and Coop (2024) and Negm and Veller (2026) (see equation (25) in the latter), we account for both these effects by assuming that each trait locus still evolves as if under underdominant selection, but with an effective selection coefficient:


(15) $$s_{i,\mathrm{eff}}\approx s_{i}{\left(\frac{V_{W}}{V_{W}^{\left(gen\right)}}\right)}^{2}\left(\frac{V_{s}}{V_{W}+V_{s}}\right)$$

The factor ${\left(\frac{V_{W}}{V_{W}^{\left(gen\right)}}\right)}^{2}$ captures the effect of negative LD between trait loci, while the factor $\left(\frac{V_{s}}{V_{W}+V_{s}}\right)$ corrects for the effect of nonnegligible $V_{W}/V_{s}$ on direct selection at the trait locus. From equation (14), it follows that $s_{i,\mathrm{eff}}/s_{i}$ depends only on $V_{W}^{\left(gen\right)}/V_{s}$.

Next, consider how migration contributes to LD between trait loci. Since all demes are adapted to the same optimum, migrants are phenotypically very similar to residents but may bear distinct genetic combinations, especially at low levels of migration, when different demes carry alternative minor alleles. Mating between migrants and residents produces F1 hybrids which are phenotypically similar to both parents but can have much higher heterozygosity. Descendants of these F1s (e.g. back-cross individuals) thus tend to be phenotypically more variable and have loweraverage fitness than the descendants of residents. This reduces the long-term contribution of migrant individuals to the gene pool and generates indirect selection against immigrant alleles due to their association with less fit genetic backgrounds (which deviate more than average from the trait optimum). Following Sachdeva (2022) and Surendranadh and Sachdeva (2025), we account for this by assuming that the effect of migration on allele frequencies is still described by equations (6) and (7), but with *m* replaced by an effective migration rate $m_{\mathrm{eff}}$. In the Supplementary information, we show that under the assumptions of our model:


(16) $$m_{\mathrm{eff}}\;\approx \;m\;\exp\;\left(-\frac{1}{3}\frac{V_{B}^{\left(gen\right)}}{V_{s}}\right)$$

where $V_{B}^{\left(gen\right)}=V_{T}^{\left(gen\right)}-V_{W}^{\left(gen\right)}$, is the among-deme genic variance (see Supplementary equation S.24). Thus, the reduction in effective migration rates is strongest when $V_{B}^{\left(gen\right)}$ is highest, i.e. when demes are nearly fixed for alternative alleles at trait loci. See also Veller and Simons (2026) for a related derivation of $m_{\mathrm{eff}}$ in a two-deme setting.

We now have a system of equations for the effective parameters $\left\{s_{i,\text{eff}}\right\}$ and $m_{\mathrm{eff}}$, and the variance components $V_{W}^{\left(gen\right)}$ and $V_{B}^{\left(gen\right)}$, which can be solved self-consistently. Technically, this is best done using an iterative process, where we start with an initial “guess” for $\left\{{\bar{\;p}}_{i}\right\}$, then obtain the various genic variances by integrating over $\psi [p\;|\;{\bar{p}}_{i}]$, and substitute these in equations (14)–(16) to compute $\left\{s_{i,\mathrm{eff}}\right\}$ and $m_{\mathrm{eff}}$. Finally, we substitute these values of $\left\{s_{i,\text{eff}}\right\}$ for $s_{i}$ and $m_{\mathrm{eff}}$ for *m* in equation (7) to solve for the new $\left\{{\bar{\;p}}_{i}\right\}$. These steps are iterated until $\left\{s_{i,\text{eff}}\right\}$, $\left\{{\bar{p}}_{i}\right\}$, and $m_{\mathrm{eff}}$ converge to fixed values. For the various cases that we have tested, this iterative procedure converges to a unique solution (for $\left\{s_{i,\mathrm{eff}}\right\}$, $\left\{{\bar{p}}_{i}\right\}$, and $m_{\mathrm{eff}}$) regardless of the choice of initial $\left\{{\bar{p}}_{i}\right\}$, even though convergence is not guaranteed in theory. We refer to the predictions obtained in this way as LD predictions, since they capture the effect of LD via effective parameters.

### Variance among demes and total variance across the full population

So far, we have focused on the genic and genetic variance within demes. In this section, we develop analytical approximations for $V_{B}$, the genetic variance among demes, which is defined as: $V_{B}=\frac{1}{D}\sum_{\;j=1}^{D}({\bar{z}}_{j}-\bar{z}{)}^{2}$, where ${\bar{z}}_{j}$ is the trait mean in the jth deme and $\bar{z}$ the trait mean across the entire population. This allows us to also calculate the total genetic variance $V_{T}=V_{W}+V_{B}$.

Calculating $V_{B}$ requires us to first find $\psi [{\bar{z}}_{j}\;|\;\bar{z}]$, the distribution of the trait mean in any (say, the jth) deme, conditional on the trait mean across the full population. Following Barton and Rouhani (1993), the expected rate of change of the trait mean in deme *j* can be written as:


(17) $$\frac{\partial {\bar{z}}_{j}}{\partial t}=V_{W,j}\frac{\partial \log\;{\bar{W}}_{j}}{\partial {\bar{z}}_{j}}+m(\bar{z}-{\bar{z}}_{j})$$

The first term describes the effects of selection on the trait mean, under the assumption that the trait value distribution within any deme has a negligible third moment (skew). The second term describes the effect of migration, which tends to pull the trait mean within any deme toward the population average. Equation (17) neglects the effect of mutation on trait means, which is justified as long as there is no mutational bias, and the trait optimum is at the center of the phenotypic range.

Using equation (17), together with the fact that mean squared fluctuations in trait mean per generation (due to genetic drift) go as $V_{W,j}/N$, we can write down the following expression for $\psi [{\bar{z}}_{j}\;|\;\bar{z}]$ under the diffusion approximation (see also Barton and Rouhani 1993):


(18) $$\psi [{\bar{z}}_{j}\;|\;\bar{z}]\propto \exp\;\left(-\frac{N({\bar{z}}_{j}-z_{\mathrm{opt}}{)}^{2}}{V_{s}}-\frac{Nm({\bar{z}}_{j}-\bar{z}{)}^{2}}{V_{W,j}}\right).$$

If the number of demes is large enough that fluctuations in $\bar{z}$ are negligible (so that $\bar{z}$ is deterministically equal to $z_{\mathrm{opt}}$), then the trait mean within any deme is normally distributed with mean $\bar{z}=z_{\mathrm{opt}}$ and variance $V_{B}$ given by:


(19) $$V_{B}=\frac{V_{W}}{2Nm+2N(V_{W}/V_{s})}.$$

Note that in the limit of no migration (i.e. as Nm→0), equation (19) reduces to $V_{B}=V_{s}/(2N)$, which is the predicted variance of the trait mean in a single population subject to stabilizing selection (Lande 1976). In the absence of selection, (i.e. as $V_{s}\to \infty$), equation (19) reduces to: $V_{B}=V_{W}/(2Nm)=\left(\frac{2F_{ST}}{1-F_{ST}}\right)V_{W}$, which is the neutral expectation (Whitlock 1999).

Equation (19) can now be used to predict $Q_{ST}$, which is defined as the ratio of the among-deme variance to the total genetic variance expected in a hypothetical panmictic population with the same allele frequencies as the subdivided population (Spitze 1993). $Q_{ST}$ is a measure of QG differentiation between populations, and in our model is given by:


(20) $$Q_{ST}=\frac{V_{B}}{V_{B}+2V_{W}}=\frac{1}{1+4Nm+4N(V_{W}/V_{s})},$$

We can also use our approximations to predict the total genetic variance across the full population using $V_{T}=V_{W}+V_{B}$. In general, the total genetic variance $V_{T}$ will differ from the genic variance $V_{T}^{\left(gen\right)}$ because of LD between trait loci as well as deviations from Hardy–Weinberg equilibrium (across the population as a whole). Thus, comparing $V_{T}$ and $V_{T}^{\left(gen\right)}$ provides an estimate of the combined strength of intra- and inter-locus statistical associations, which, in turn, reflect the combined effects of population structure and stabilizing selection. For the special case of equal-effect trait loci, we have: $V_{T}^{\left(gen\right)}=V_{W}^{\left(gen\right)}/(1-F_{ST})$, where $1-F_{ST}$ is measured as the ratio of the average within-deme heterozygosity averaged across all trait loci to the average total heterozygosity (see also Weir and Cockerham 1984). This gives:


(21) $$\frac{V_{T}}{V_{T}^{\left(gen\right)}}\;=\;(1+Q_{ST})\;\left(\frac{1-F_{ST}}{1-Q_{ST}}\right)\frac{V_{W}}{V_{W}^{\left(gen\right)}}$$

Note that for a neutrally evolving trait, $Q_{ST}=F_{ST}$, and $V_{W}=V_{W}^{\left(gen\right)}$, so that $V_{T}=(1+F_{ST})V_{T}^{\left(gen\right)}$ (Wright 1949; Whitlock 1999). Thus, neutral population structure *increases* genetic variance relative to genic variance by creating a deficit of heterozygotes across the population as a whole, which causes per-locus contributions to $V_{T}$ to be more extreme than one would expect in a panmictic population with the same allele frequencies. By contrast, stabilizing selection *reduces* genetic variance relative to genic variance by “locking in” alleles with opposite effects on trait value, thus creating negative LD between trait loci and reducing $V_{W}$ and (especially) $V_{B}$. Equation (21) describes how these two opposing effects together shape genetic variance. In particular, it suggests that genetic variance should be significantly lower than genic variance (i.e. the effect of negative LD between trait loci should dominate), provided $F_{ST}$ at trait loci is high and $Q_{ST}$ is low (see also Le Corre and Kremer 2003).

The arguments outlined in this section (and in deriving equation (14) in the previous section) are framed purely in terms of trait means and variances. We thus refer to equations (14), (19), and (20) as QG predictions. As before, we can build upon these to account for the effects of LD within demes using effective parameters. This is accomplished by replacing *m* by $m_{\mathrm{eff}}$ in equations (19) and (20), and by using $m_{\mathrm{eff}}$ and $\left\{s_{i,\text{eff}}\right\}$ in equation (7) when computing $V_{W}^{\left(gen\right)}$ (which then predicts $V_{W}$). We refer to these as QG(eff) predictions.

### Simulations

We test analytical predictions by comparing against discrete-generation, individual-based simulations which track multi-locus genotypes of all diploid individuals across all demes. In each generation, the life cycle consists of mutation, followed by selection and mating within demes, which is then followed by migration. We perform two kinds of simulations: the first with underdominant selection on a single locus (Fig. 1), and the second with stabilizing selection on an additive trait influenced by *L* loci, where effect sizes at trait loci are either all equal (as in Fig. 2) or drawn from a distribution (Fig. 3). See also the Model description above.

**Fig. 1.**
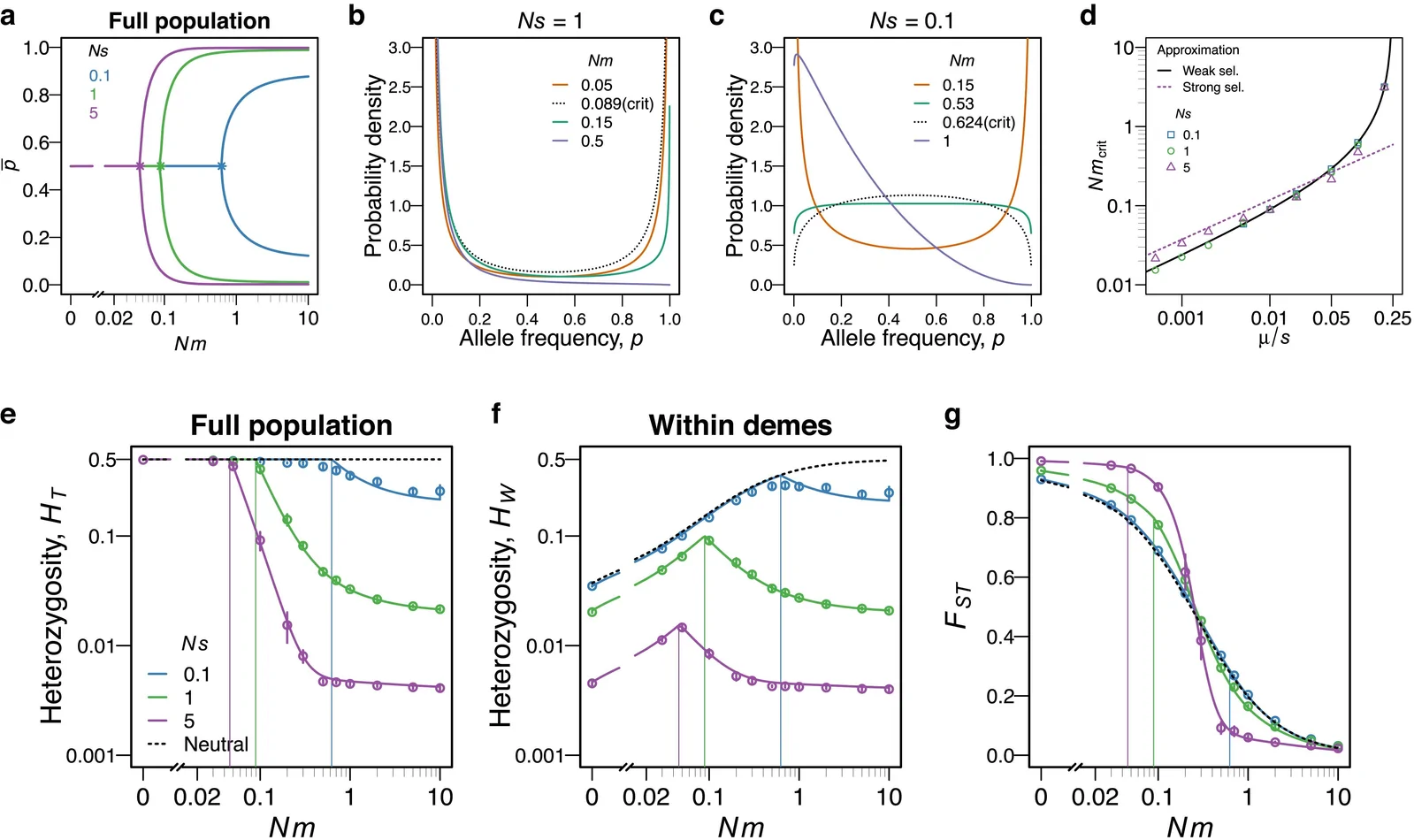
Genetic diversity at a single underdominant locus under the island model. a) Mean allele frequency across the full population, $\bar{\;p}$, as a function of *Nm* for different *Ns* values (colors), at equilibrium. There is a critical migration threshold $Nm_{\mathrm{crit}}$ (marked by a *), such that there is only one stable equilibrium $\bar{\;p}=1/2$ for $Nm<Nm_{\mathrm{crit}}$, but two alternative equilibria ${\bar{\;p}}_{+}>1/2$ and ${\bar{\;p}}_{-}<1/2$ for $Nm>Nm_{\mathrm{crit}}$. b) and c) Equilibrium probability density, $\Phi [p|\bar{p}]$, of the allele frequency *p* in a single deme, for different values of *Nm* (colors), including $Nm_{\mathrm{crit}}$ (dashed black curve), for Nμ=0.01 and scaled selection coefficient b) Ns=1 or c) Ns=0.1. The probability density is symmetric around p=1/2 if $Nm<Nm_{\mathrm{crit}}$ but biased toward $\bar{\;p}$ (which we assume to be equal to ${\bar{\;p}}_{-}$) if $Nm>Nm_{\mathrm{crit}}$. All solid lines in a)–c) are obtained from the diffusion approximation (equation 7). d) The scaled critical migration rate $Nm_{\mathrm{crit}}$ as a function of μ/s for Nμ=0.01, and different values of *Ns*. Symbols show $Nm_{\mathrm{crit}}$ as obtained from the diffusion approximation; the solid line shows the “weak selection” approximation for $Nm_{\mathrm{crit}}$ (Supplementary equation S.4) which is independent of *Ns* (for a given μ/s); the dashed line shows the “strong selection” approximation for $Nm_{\mathrm{crit}}$ (Supplementary equation S.11) for Ns=5. e) Total heterozygosity across the full population, $H_{T}=2\bar{p}\;\bar{q}$, (f) Within-deme heterozygosity, $H_{W}=E[2pq]$ and g) expected $F_{ST}$ at selected locus as a function of *Nm*, for different values of *Ns*, and Nμ=0.01. Solid lines show predictions of the diffusion approximation (equation 7); dashed lines represents the neutral prediction, and circles show results of individual-based simulations with D=100 demes and N=200 diploids per deme, where each simulation is run for $5\times 10^{5}$ generations. Each circle shows the mean obtained by averaging over 10 simulation replicates and over the last $10^{4}$ generations (at intervals of $10^{3}$ generations) within each replicate; error bars show 1.96 times the standard error (SE). For reference, we also indicate $Nm_{\mathrm{crit}}$ thresholds (as predicted by the diffusion approximation) using vertical lines in panels e), f), and g).

**Fig. 2.**
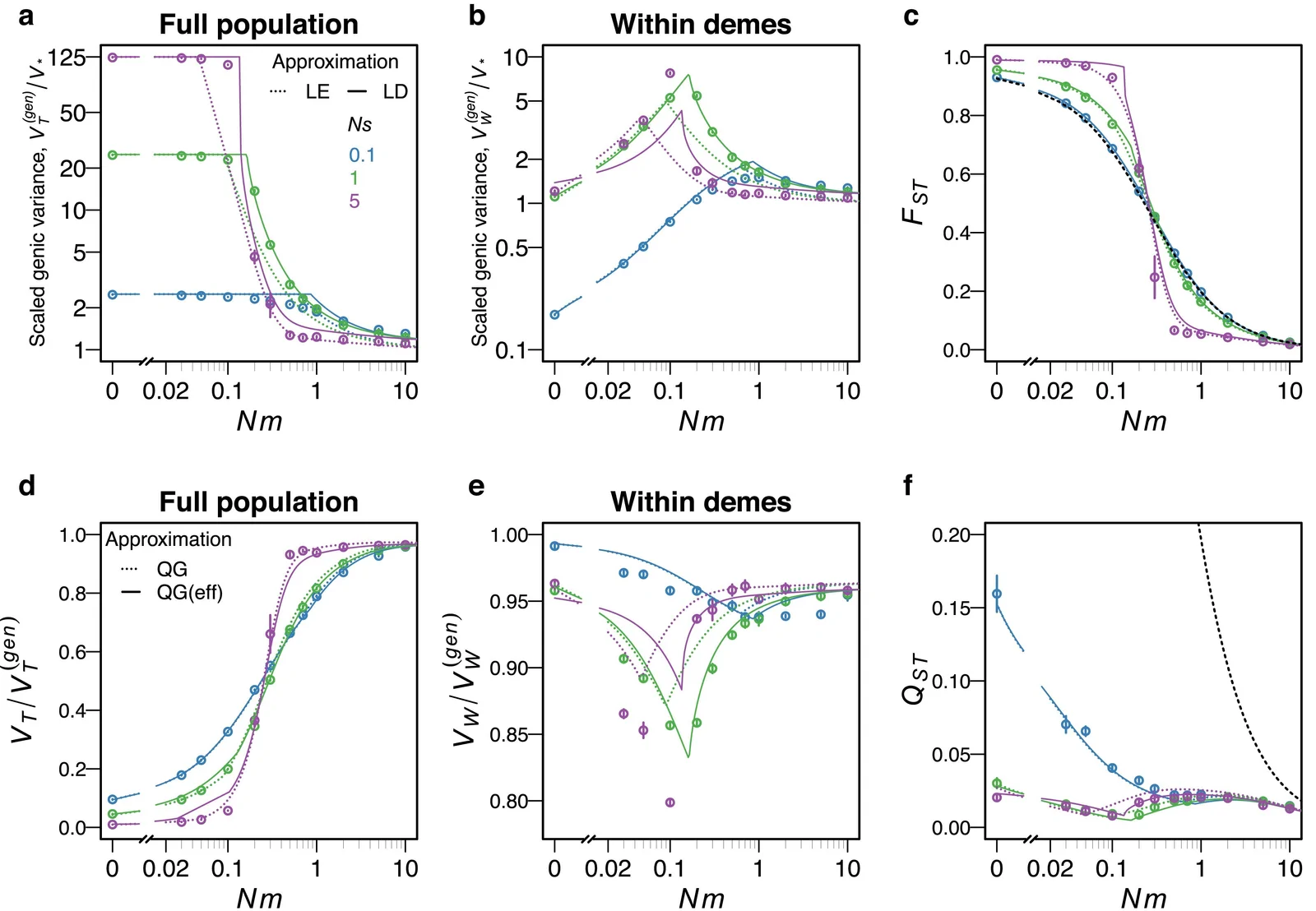
Effect of stabilizing selection and population structure on genic variance, genetic variance, $F_{ST}$ and $Q_{ST}$, for an additive trait influenced by equal-effect loci. The expected a) total genic variance across the full population, $V_{T}^{\left(gen\right)}/V_{*}$, and b) within-deme genic variance, $V_{W}^{\left(gen\right)}/V_{*}$, as a function of *Nm*, the average number of migrants per deme per generation. Genic variances are scaled by $V_{*}=4\mu LV_{s}$, the variance in a large panmictic population at deterministic mutation-selection balance (neglecting LD). c) Average $F_{ST}$ across all trait loci vs. *Nm*, d) ratio of the genetic variance to the genic variance in the population as a whole, $V_{T}/V_{T}^{\left(gen\right)}$, and e) the corresponding ratio within demes, $V_{W}/V_{W}^{\left(gen\right)}$, as a function of *Nm*. f) $Q_{ST}$ vs. *Nm*. The different colors show results for different values of *Ns*, the scaled selection coefficient per locus, where $s=\frac{\alpha^{2}}{2V_{s}}$ and α is the effect size of the locus. For a trait with equal-effect loci, the scaled variances can be expressed as: $\frac{V_{T}}{V_{*}}=\frac{Ns}{N\mu }\bar{p}\;\bar{q}$ and $\frac{V_{W}}{V_{*}}=\frac{Ns}{N\mu }E[pq]$. Symbols show results of individual-based simulations with D=100 demes and N=200 diploids per deme that express an additive trait influenced by L=200 equal-effect loci, with scaled mutation rate Nμ=0.01 per locus. Each circle shows the mean obtained by averaging over 10 simulation replicates and over the last $10^{4}$ generations (at intervals of $5\times 10^{3}$ generations) within each replicate; error bars show 1.96 times the standard error. Simulations are run for $10^{5}$ generations, except those with Ns=5 and Nm≤0.3, which are run longer (for $5\times 10^{5}$ generations). The dashed lines in a)–c) show LE predictions based on the diffusion approximation for an underdominant locus (and are the same as the predictions in Fig. 1e–g up to a constant scaling factor). Solid lines in a)–c) show LD predictions that account for the effect of LD on heterozygosity at trait loci using effective parameters. Dashed lines in d)–f) show QG predictions based on equations (14), (19), and (21) together with single-locus theory for the genic variances; solid lines show ${\text{QG}}_{\mathrm{eff}}$ predictions that use effective parameters.

**Fig. 3.**
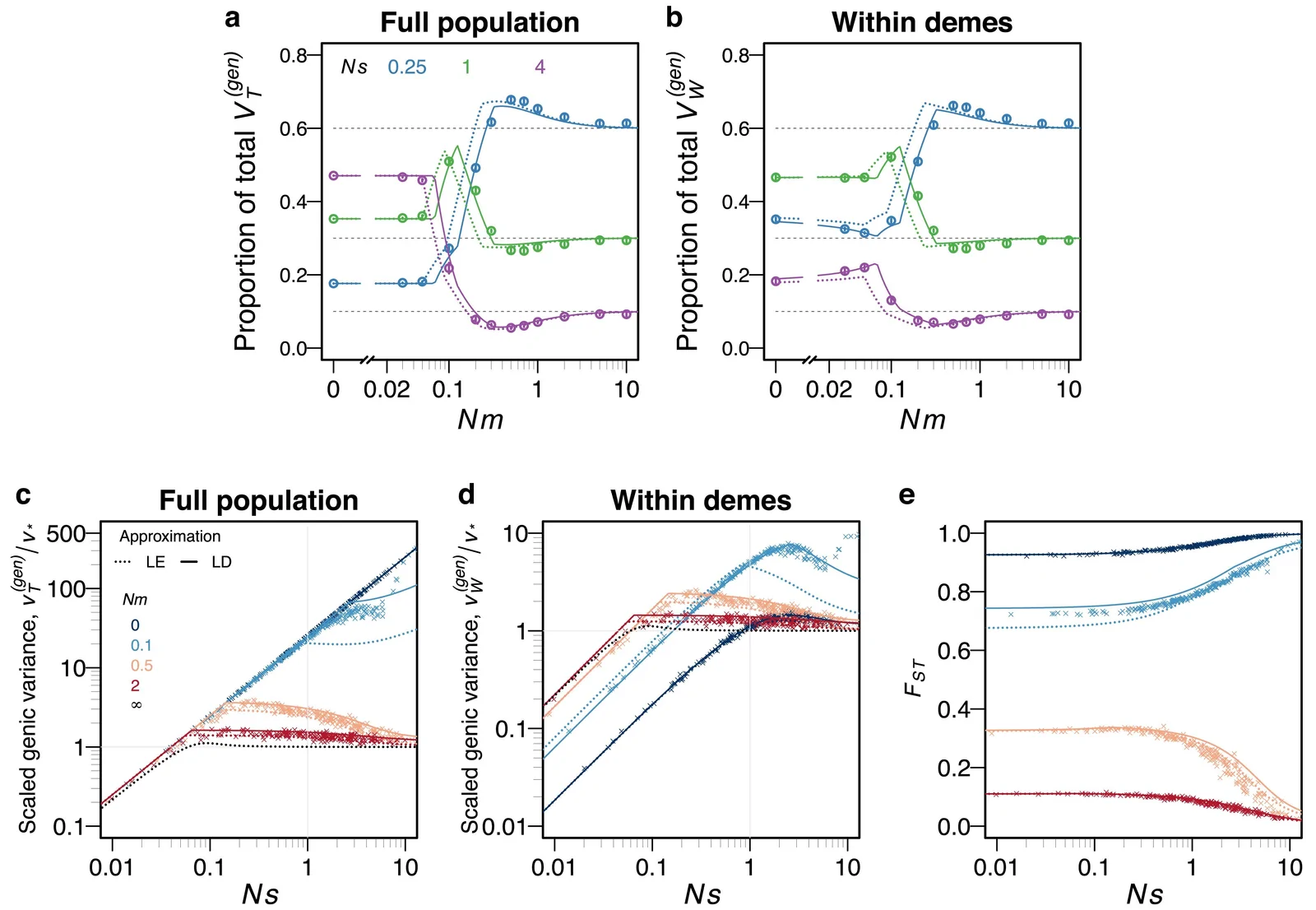
Variation in traits influenced by loci of unequal effect. a)–b) Proportion of a) total genic variance and b) within-deme genic variance contributed by small-effect (Ns=0.25), medium-effect (Ns=1), and large-effect (Ns=4) loci, for a trait influenced by L=200 unlinked loci, of which 60%, 30%, and 10% have small, medium and large effects respectively. The three colors correspond to the three different effect sizes. c)–e) Expected contribution of each trait locus to c) within-deme genic variance and d) total genic variance across the full population, and e) expected $F_{ST}$ at each trait locus, as a function of the scaled selection coefficient (*Ns*) of the locus, for a trait influenced by L=200 loci with effect sizes drawn from an exponential distribution with $N\;\bar{s}=2$. Here, per-locus variance contributions are scaled by the deterministic expectation, $v_{*}=4\mu V_{s}$. Different colors correspond to different migration rates; dotted black line shows LE prediction in the limit Nm→∞, i.e. for a single *panmictic* population with population size $N_{\mathrm{tot}}=ND$. Circles (in a)–b)) and points (in c)–e)) show results of individual-based simulations with D=100 demes and N=200 diploid individuals per deme, with scaled mutation rate Nμ=0.01 per locus. Each circle/point shows the mean obtained by averaging over 10 simulation replicates and over the last $10^{4}$ generations (at intervals of $5\times 10^{3}$ generations) within each replicate. The dashed lines show LE predictions based on the diffusion approximation for an underdominant locus; solid lines show LD predictions using effective parameters. In c)–e) and for Nm=0.1, loci with the largest *Ns* values have not yet equilibrated in simulations run for $5\times 10^{5}$ generations and initialized with allele frequencies at each locus in each deme drawn independently from a beta distribution Beta[4Nμ,4Nμ].

Simulations are performed with 2 different kinds of initial conditions—we either draw allele frequencies at each locus *in each deme* independently from a beta distribution Beta[4Nμ,4Nμ], or first draw the mean allele frequencies $\left\{{\bar{\;p}}_{1},{\bar{\;p}}_{2},\ldots ,{\bar{\;p}}_{L}\right\}$ at each locus from the beta distribution and then set the allele frequencies in individual demes to be equal to the respective ${\bar{\;p}}_{i}$. As shown in Supplementary Fig. S5, the time taken for the population to reach equilibrium can be quite different, depending on the initial conditions (especially at low migration rates), even though the equilibrium state itself is independent of initial conditions. Simulations are run for D=100 demes and N=200 individuals per deme, and a maximum of $10^{6}$ generations, which is found to be sufficient for equilibration in all cases (including very weak migration), with equilibration being faster at larger migration rates (see also Supplementary Fig. S5).

**Parameters and quantities used to define the model**

*D*  Number of demes.
*N*  Number of diploid individuals per deme.
*L*  Number of loci influencing additive trait.

$z_{i,j}$ Trait value of individual *i* in deme *j*.

$\alpha_{i}$ Effect size at locus *i*. Genotypes 00,01,11 at locus *i* make additive contributions $-\alpha_{i},0,\alpha_{i}$ respectively to trait value.

$W(z)=e^{-\frac{(z-z_{\mathrm{opt}}{)}^{2}}{2V_{s}}}$ Fitness of an individual with trait value *z*, where $z_{\mathrm{opt}}$ is the optimal trait value, and $\sqrt{V_{s}}$ is the width of the fitness function.

$s_{i}=\frac{\alpha_{i}^{2}}{2V_{s}}$ Strength of underdominant selection at locus *i*.
*μ*  Mutation rate per allele per generation.
*m*  Migration rate per individual per generation.

**Measures of genetic diversity**

$p_{i,j}$ and $q_{i,j}=1-p_{i,j}$  Frequencies of trait-increasing and trait-decreasing alleles at locus *i* in deme *j*.

${\bar{\;p}}_{i}=\frac{1}{D}\sum_{\;j=1}^{D}p_{i,j}$ and ${\bar{q}}_{i}=1-{\bar{\;p}}_{i}$  Mean frequencies of trait-increasing and trait-decreasing alleles at locus *i* across the full population.

${\bar{z}}_{j}=\sum_{i=1}^{L}\alpha_{j}(2p_{i,j}-1)$ and $\bar{z}=\sum_{i=1}^{L}\alpha_{j}(2{\bar{\;p}}_{i}-1)$  Mean trait value in deme *j* and mean trait value across the full population.

$H_{W}^{\left(i\right)}=\frac{1}{D}\sum_{\;j=1}^{D}2p_{i,j}q_{i,j}$ and $H_{T}^{\left(i\right)}=2{\bar{\;p}}_{i}{\bar{q}}_{i}$  Within-deme heterozygosity (averaged over all demes) and total heterozygosity across the full population at locus *i*.

$V_{W}^{\left(gen\right)}=\frac{1}{D}\sum_{\;j=1}^{D}\sum_{i=1}^{L}2\alpha_{i}^{2}p_{i,j}q_{i,j}$ , $V_{B}^{\left(gen\right)}=\frac{1}{D}\sum_{\;j=1}^{D}\sum_{i=1}^{L}2\alpha_{i}^{2}(p_{i,j}-{\bar{\;p}}_{i}{)}^{2}$, and $V_{T}^{\left(gen\right)}=\sum_{i=1}^{L}2\alpha_{i}^{2}{\bar{\;p}}_{i}{\bar{q}}_{i}$  Within-deme genic variance (averaged across all demes), among-deme genic variance, and total genic variance across the full population; $V_{T}^{\left(gen\right)}=V_{W}^{\left(gen\right)}+V_{B}^{\left(gen\right)}$. Lower-case $v_{W}^{\left(gen\right)}$, $v_{B}^{\left(gen\right)}$ and $v_{T}^{\left(gen\right)}$ indicate genic variances for a given locus.

$V_{W}=\frac{1}{DN}\sum_{\;j=1}^{D}\sum_{i=1}^{N}(z_{i,j}-{\bar{z}}_{j}{)}^{2}$ , $V_{B}=\frac{1}{D}\sum_{\;j=1}^{D}({\bar{z}}_{\;j}-\bar{z}{)}^{2}$, and $V_{T}=\frac{1}{DN}\sum_{\;j=1}^{D}\sum_{i=1}^{N}(z_{i,j}-\bar{z}{)}^{2}$  Within-deme genetic variance (averaged across all demes), among-deme genetic variance, and total genetic variance across the full population; $V_{T}=V_{W}+V_{B}$.

$F_{ST}=\frac{H_{T}-H_{W}}{H_{T}}$ Proportion of total heterozygosity (at a given locus) explained by variance in allele frequencies across demes.

$Q_{ST}=\frac{V_{B}}{V_{B}+2V_{W}}$ Relative measure of quantitative genetic differentiation between demes, analogous to $F_{ST}$. For a neutrally evolving trait, $Q_{ST}=F_{ST}$.

**Scaled and composite parameters**

Nμ,Nm,Ns Mutation rate, migration rate, and selection coefficient scaled by population size per deme *N*.

$V_{*}=4\mu LV_{s},\;v_{*}=4\mu V_{s}$ Expected genic variance for a trait under stabilizing selection or at a single trait locus in a panmictic population at deterministic mutation-selection balance.

$s_{\mathrm{eff}}\approx s{\left(\frac{V_{W}}{V_{W}^{\left(gen\right)}}\right)}^{2}\left(\frac{V_{s}}{V_{s}+V_{W}}\right)$ Effective selection coefficient used to approximate the effects of LD due to stabilizing selection within demes.

$m_{\mathrm{eff}}\approx \;m\;\exp\;\left(-\frac{1}{3}\frac{V_{B}^{\left(gen\right)}}{V_{s}}\right)$ Effective migration rate used to approximate the effects of LD due to migration between demes.

## Results

As discussed above, stabilizing selection on a polygenic trait essentially manifests as underdominant selection on individual trait loci when the trait mean is at the trait optimum. Thus, as a precursor to analyzing the polygenic model, we first consider the behavior of a single locus under underdominant selection in the infinite-island setting. We then build upon this to investigate how variation in the polygenic trait—at individual loci (genic variance) and in trait values (genetic variance)—is partitioned within and among demes. This requires us to go beyond single-locus theory and also account for LD between trait loci, which we do via effective selection and migration parameters (see Methods). Finally, we investigate questions of direct empirical relevance: how many and what kind of loci make atypically large contributions to trait variance, and to what extent such outliers are shared across demes. Throughout, we compare the results of individual-based simulations with LE and LD predictions, thus clarifying to what extent LD influences patterns of variation and whether we can describe its effects succinctly in terms of a handful of effective parameters.

### Single locus under underdominant selection

Consider a single biallelic locus under underdominant selection (Fig. 1). If selection, migration and mutation are sufficiently weak (s,m,μ≪1), and if the population size per deme is large (N≳50), then the allele frequency distribution at migration-mutation-selection-drift equilibrium depends only on the scaled parameters *Ns*, *Nm* and Nμ via equation (7). The population exhibits two qualitatively different types of equilibrium states, separated by a critical (scaled) migration rate $Nm_{\mathrm{crit}}$ (Fig. 1a; see also Barton and Rouhani 1993).

At low migration rates (i.e. for $Nm\leq Nm_{\mathrm{crit}}$), alternative alleles increase to high frequencies in different demes, leading to high levels of polymorphism in the population as a whole. The mean allele frequency across the full population is $\bar{\;p}=1/2$, and the total population-wide heterozygosity is $H_{T}=2\bar{p}(1-\bar{p})=1/2$, regardless of *Nm* (Fig. 1e). The allele frequency distribution within demes is symmetric around 1/2 (Fig. 1b,c); it is U-shaped if selection is strong, i.e. $Nm<min(1/2,Nm_{\mathrm{crit}})$ (neglecting O(Nμ) terms), but inverted U-shaped if selection is weak, i.e. $1/2<Nm<Nm_{\mathrm{crit}}$ (see, e.g. green curve in Fig. 1c).

By contrast, for $Nm>Nm_{\mathrm{crit}}$, the evolution of different demes is strongly coupled, and the *same* allele reaches high frequency in the majority of demes. The mean frequency $\bar{\;p}$ across the full population evolves toward one of two possible equilibrium values, ${\bar{p}}_{-}<\frac{1}{2}$ or ${\bar{p}}_{+}>\frac{1}{2}$. The total heterozygosity $H_{T}$ is thus less than $\frac{1}{2}$, and decreases with increasing migration, approaching 2(μ/s) (the expected heterozygosity in a large panmictic population) at large values of *Nm* (Fig. 1e). The allele frequency distribution within demes is no longer symmetric about $p=\frac{1}{2}$. Instead, it is unimodal and concentrated around $\bar{p}$ if $Nm≳\max(Nm_{\mathrm{crit}},\frac{1}{4})$ or U-shaped with a strong skew toward $\bar{p}$ if $Nm_{\mathrm{crit}}<Nm≲\frac{1}{4}$ (see, e.g. green curve in Fig. 1b).

In Supplementary Sec. SI.a, we derive approximate expressions for the critical migration rate and show that $Nm_{\mathrm{crit}}$ depends primarily on μ/s, and follows:


(22) $$Nm_{\mathrm{crit}}\approx \frac{\sqrt{\left(\mu /s{)}^{2}+2(\mu /s\right)}+3(\mu /s)}{2(1-4(\mu /s))}$$

for Ns≲1 and μ/s<1/4 (solid black line in Fig. 1d). Thus, as selection becomes stronger (and μ/s smaller), lower levels of migration are sufficient to couple evolution across demes. Note that we restrict ourselves to μ/s<1/4, since at higher mutation rates, underdominant selection is overwhelmed by mutation, driving allele frequencies to p=1/2.

We also track $H_{T}$ and $H_{W}$, the total and within-deme heterozygosity, as well as $F_{ST}$ at the selected locus, and compare analytical predictions based on equation (7) with individual-based simulations of D=100 demes and N=200 individuals per deme (Fig. 1e–g). Simulations are conducted for 3 different selection strengths (Ns=0.1,1,5)—representing weak, intermediate, and strong selection relative to local drift. The corresponding predictions for $Nm_{\mathrm{crit}}$ are 0.624, 0.089, and 0.046 (depicted by vertical lines in Fig. 1e,f). These figures show close agreement between simulations and theory. Slight deviations for weak selection and $Nm≃Nm_{\mathrm{crit}}$ are due to the relatively modest total population size $N_{tot}=ND$ (see Supplementary Sec. SI.c), and vanish as the number of demes, and consequently $N_{tot}$, increases (Supplementary Fig. S2).

Figure 1e,f shows that the effect of migration on heterozygosities differs qualitatively depending on whether *Nm* is above or below the critical migration threshold $Nm_{\mathrm{crit}}$. As described above, both $H_{T}$ and $H_{W}$ decrease with increasing migration for $Nm>Nm_{\mathrm{crit}}$, but are unchanged (in the case of $H_{T}$) or increase (in the case of $H_{W}$) with *Nm*, if $Nm<Nm_{\mathrm{crit}}$. In particular, $H_{W}$, the heterozygosity within demes, is maximized at $Nm∼Nm_{\mathrm{crit}}$, being lower at both weaker and stronger levels of migration—when either individual demes or the population as a whole are close to fixing one of the alleles. Thus, while strong population subdivision allows high genetic variation to be maintained across the full population, intermediate levels of subdivision are most effective at maintaining genetic variation within local demes.

Interestingly, $Nm_{\mathrm{crit}}$ is not associated with any qualitative change in the behavior of $F_{ST}$, which decreases smoothly with increasing migration (Fig. 1g). For $Nm<Nm_{\mathrm{crit}}$, this decrease is purely due to the increase in $H_{W}$ (since $H_{T}$ is equal to 1/2, regardless of *Nm*). By contrast, for $Nm>Nm_{\mathrm{crit}}$, the decrease in $F_{ST}$ primarily reflects changes in $H_{T}$, which decreases much more sharply than $H_{W}$ with increasing *Nm* (compare, e.g. Fig. 1e,f).

Figure 1g also shows how the strength of underdominant selection influences $F_{ST}$. Under weak selection (Ns=0.1), $F_{ST}$ is almost indistinguishable from the neutral expectation (dashed black line), regardless of *Nm*. In contrast, for stronger selection (Ns=1 or 5), $F_{ST}$ at the selected locus is higher than neutral $F_{ST}$ for 4Nm≲1, but then drops below it for 4Nm≳1. To better understand these observations, we develop simpler analytical approximations (described in Supplementary Sec. SI.a; see also Supplementary Fig. S1) for the weak selection (Nμ≪Ns≲1) and strong selection (Nμ≪1≲Ns) limits. These show that $F_{ST}$ goes from slightly above the neutral prediction to slightly below it at 4Nm=1+O(μ/s) for any locus with $4Nm_{\mathrm{crit}}<1$, which translates into μ/s≲1/24 (see equation (22)), i.e. moderate or strong underdominant selection.

Interestingly, for such loci, the shape of the allele frequency distribution also changes qualitatively—from asymmetric U-shaped (a minority of demes close to fixing the alternative allele) to single-peaked (same allele common across all demes)—at a migration threshold 4Nm=1+O(μ/s). This threshold is very close (but not exactly equal) to the threshold at which $F_{ST}$ falls below the neutral prediction. Indeed, the two thresholds coincide as μ/s→0. More generally, while increasing selection reduces both $H_{W}$ and $H_{T}$, its effect on the total heterozygosity, $H_{T}$, is weaker if 4Nm≲1, i.e. if at least some demes remain locally fixed for the alternative allele. Thus, in this regime, $F_{ST}$ increases with increasing selection.

### Polygenic trait under stabilizing selection

Building upon the single-locus analysis of the previous section, we now ask: how much variation is maintained in a polygenic trait under stabilizing selection toward a uniform optimum? Stabilizing selection has two main effects—it pushes the trait mean toward the trait optimum, and it reduces the trait variance. The first effect is typically quite strong, causing ${\bar{z}}_{i}$, the trait mean within any deme *i*, to change as: $\frac{d\;{\bar{z}}_{i}}{dt}=-\frac{V_{W}}{V_{s}}({\bar{z}}_{i}-z_{\mathrm{opt}})$, where $V_{W}$ is the within-deme trait variance. Thus, trait means tend to rapidly approach the trait optimum, so that ${\bar{z}}_{i}\approx z_{\mathrm{opt}}$ in expectation, and $\text{Var}({\bar{z}}_{i})=V_{B}$ is small ($≲V_{s}/(2N)$), regardless of the genetic basis of the trait, provided there is no mutation bias or migration from differently adapted populations.

In contrast, the effect of stabilizing selection on trait variance is mediated by the effect sizes of trait loci. Stabilizing selection reduces trait variance in two ways: first, by disfavoring polymorphism at individual trait loci, and second, by disfavoring multi-locus combinations with a higher than average number of alleles with the same (say, positive) effect on trait value. Selection against polymorphism causes a trait locus with effect size *α* to behave as an underdominant locus with selection coefficient $s=\frac{\alpha^{2}}{2V_{s}}$ (Robertson 1956). Selection against multi-locus combinations results in negative fitness epistasis between trait-increasing (or trait-decreasing) alleles, which generates negative LD between trait loci. This kind of negative LD, in turn, has three consequences. First, it causes the genetic variance (i.e. variance in trait values across individuals) to be lower than the genic variance, both within demes and across the full population. Second, it effectively weakens underdominant selection at each locus, thus slightly increasing heterozygosity at trait loci and the genic variance. Third, it reduces the long-term genetic contribution of immigrants to variation within demes, especially under conditions of low gene flow when different demes support different multi-locus combinations.

Below, we compare simulation results with analytical predictions that either neglect the effects of LD on genic variance (LE predictions), or account for it via effective parameters (LD predictions). As detailed in the Methods, the LE prediction treats each trait locus as evolving independently under underdominant selection, allowing us to use the single-locus analysis of the previous section to predict genic variances. The LD predictions then build upon this by assuming that LD due to stabilizing selection essentially alters the effective selection coefficient $s_{\mathrm{eff}}$ at each trait locus by a factor that depends on the within-deme genic variance $V_{W}^{\left(gen\right)}/V_{s}$, while LD due to migration changes the effective migration rate $m_{\mathrm{eff}}$ by a factor that depends on the among-deme genic variance $V_{B}^{\left(gen\right)}/V_{s}$.

The main question we address here is: how much more trait variation can be maintained in a structured population compared to an unstructured one? To this end, we always scale trait variances by $V_{*}=4\mu LV_{s}$, the expected genic variance in a large panmictic population under mutation-selection balance (neglecting LD). In the main text, we keep Nμ=0.01 fixed. In Supplementary Sec. SI.f, we consider other values of Nμ and show that for a given *Nm*, various quantities depend primarily on the ratio Nμ/Ns=μ/s (rather than mutation rates and selection coefficients separately).

We first consider traits influenced by *L* loci of *equal* effect size, $\alpha_{i}=\alpha$ for all *i* (and selection coefficient $s_{i}=s=\alpha^{2}/(2V_{s})$). Figure 2 shows how the genic and genetic variances change with *Nm*, the average number of migrants per deme, across the full population (Fig. 2a,d) and within demes (Fig. 2b,e). Figure 2c,f shows the effect of increasing migration on the expected $F_{ST}$ at trait loci and the expected $Q_{ST}$, both of which quantify how genetic variation (at the locus level and trait-level, respectively) is partitioned within and among demes. Symbols show results of individual-based simulations; dashed and solid lines depict LE and LD predictions respectively. The different colors correspond to different strengths of stabilizing selection, $V_{s}$. However, since $V_{s}$ only affects evolutionary dynamics via $s=\alpha^{2}/(2V_{s})$, the colors can also be thought of as showing results for traits influenced by loci of large vs. small effect.

#### Genic variances and $F_{ST}$ at trait loci

First consider how population structure affects $V_{T}^{\left(gen\right)}$, the total genic variance. Figure 2a shows that $V_{T}^{\left(gen\right)}/V_{*}$ is independent of migration for $Nm<Nm_{\mathrm{crit}}$, where $Nm_{\mathrm{crit}}$ observed in simulations is accurately predicted by our LD approximation (and is higher than the critical threshold for a single underdominant locus with $Ns=N(\frac{a^{2}}{2V_{s}})$; dashed lines). For $Nm<Nm_{\mathrm{crit}}$, the mean allele frequency in the population at any trait locus is $\bar{\;p}=1/2$, and $V_{T}^{\left(gen\right)}/V_{*}=\frac{s}{4\mu }$ (which is typically much larger than 1), so that the total genic variance in the subdivided population is much higher than that in a large panmictic population. In this parameter regime, different demes occupy different adaptive peaks, so that high levels of variation are maintained in the population as a whole. However, once *Nm* exceeds $Nm_{\mathrm{crit}}$, the same allele tends to become common across all demes at any trait locus, causing $V_{T}^{\left(gen\right)}$ to fall. For large enough migration rates, the subdivided population essentially behaves as a single panmictic population, so that $V_{T}^{\left(gen\right)}/V_{*}∼1$.

Figure 2a also shows that population structure increases $V_{T}^{\left(gen\right)}$ most strongly if *Ns* per locus (and hence, s/μ) is large; however, this effect is then restricted to very low migration rates. In contrast, for small *Ns*, the effect of population structure on trait variance is much weaker, but extends over a wider range of migration rates. Thus, the increase in trait variance in populations with (say) Nm≳1/2 is expected to be modest and entirely due to loci of small or moderate effect.

Next consider how population structure affects the within-deme genic variance, $V_{W}^{\left(gen\right)}$. Figure 2b shows that $V_{W}^{\left(gen\right)}/V_{*}$ increases with *Nm* at low levels of migration, reaches a maximum at $Nm∼Nm_{\mathrm{crit}}$, and then begins to decrease as migration increases further, thus mirroring the behavior of within-deme heterozygosity at a single underdominant locus (Fig. 1f). In fact, at $Nm∼Nm_{\mathrm{crit}}$, the genic variance within a single deme can be as much as 5 times that in a large panmictic population, provided that μ/s≲0.01. More generally, the fact that within-deme genic variance is maximized at *intermediate* levels of migration reflects the tension between two qualitatively different—“mixing” vs “homogenizing”—effects of migration. For individual demes to maintain high levels of variation, migration must be strong enough to introduce genetic variants from other demes into the focal deme (a “mixing” effect), but not so strong as to drive the same variant close to fixation across all demes (a “homogenizing” effect).

We can also quantify the relative contributions of within-deme and among-deme variation to the overall polymorphism at trait loci using $F_{ST}$ (Fig. 2c). This changes with *Nm* in essentially the same way as the $F_{ST}$ at a single underdominant locus (see also Fig. 1g). In particular, for any locus with $4Nm_{\mathrm{crit}}<1$ (i.e. μ/s≲1/24, equation 22; e.g. green and purple curves in Fig. 2c), the average $F_{ST}$ is higher (lower) than the neutral expectation (black dashed line), depending on whether 4Nm is lower (higher) than ∼1. This observation is interesting in light of previous work that predicts that spatially uniform stabilizing selection should cause trait loci to become more strongly differentiated between diverging populations than neutral loci (Latta 1998; Le Corre and Kremer 2012). Our analysis suggests that this is only true if populations evolve largely independently, with even moderate levels of migration being enough to reverse the pattern.

Figure 2a–c shows that while LE predictions (dashed lines) capture the qualitative behaviour of genic variances, predictions that account for LD using effective parameters (solid lines) are more accurate, especially for moderate selection per locus (Ns=1).

Both types of predictions deviate modestly from simulation results for very weak or strong selection. For weak selection (Ns≪1), the discrepancy between simulations and theory is a consequence of the relatively small number of demes (D=100) in simulations, which leads to large fluctuations in $\bar{\;p}$, the mean allele frequency at trait loci. As in the case of a single underdominant locus (Supplementary Fig. S2), the agreement between simulations and theory improves with increasing total population size. For strong selection (Ns≫1), theoretical predictions become less accurate for a different reason. If selection per locus is strong and the total mutational input per deme (NμL) is low, then very few trait loci are polymorphic within a deme. For instance, for Nm=1, Ns=5, and NμL=2 (in Fig. 2), only 6.4% or 1.3% (i.e. ∼13 or ∼3) of the L=200 loci are expected to have minor allele frequencies greater than 0.01 or 0.05. Thus, the basic assumptions underlying our theoretical approximations—that trait means hardly deviate from the trait optimum and that trait values are normally distributed—are likely to be violated. Based on this reasoning, we also expect predictions to become more accurate as the number of polymorphic loci increases. To test this, we simulated a single deme under stabilizing selection for different values of NμL, while holding constant the scaled parameters *Ns* and Nμ (which govern single-locus dynamics) and μL (which determines the extent of LD in the NμL→∞ limit). Simulation results indeed approach LD predictions as NμL increases (Supplementary Fig. S4).

#### Genetic variance within demes and across the entire population

We now explore how population structure and selection affect $V_{W}$ and $V_{T}$, the genetic variance within demes and across the whole population, as well as $Q_{ST}=V_{B}/(V_{B}+2V_{W})$, which can be thought of as the QG analog of $F_{ST}$ (Spitze 1993).

Figure 2d shows $V_{T}/V_{T}^{\left(gen\right)}$, the ratio of the total genetic variance to the total genic variance across the full population, as a function of *Nm* for different strengths of selection. Figure 2e shows the corresponding plot for $V_{W}/V_{W}^{\left(gen\right)}$, the ratio of within-deme genetic to genic variance. Note that these ratios are always less than 1, which means that, on average, alleles with similar (e.g. positive) effect on trait value are in negative LD with each other.

Negative LD within demes is primarily a consequence of the Bulmer effect, wherein phenotypically extreme individuals, i.e. those with a higher than average number of trait-increasing or trait-decreasing alleles, have lower fitness. This generates negative selection against combinations of such alleles, thus reducing the variance of trait values. However, this is counterbalanced in every generation by the variance released due to random mating and segregation. This so-called segregation variance is rather large (being equal to half the genic variance) when trait loci are unlinked. Thus, within demes, genetic variance is only modestly reduced relative to genic variance: $V_{W}/V_{W}^{\left(gen\right)}\approx 1-V_{W}^{\left(gen\right)}/V_{s}$ (see also equation (14)). This also suggests that the effects of LD within demes should be strongest when $Nm∼Nm_{\mathrm{crit}}$, i.e. when genic variance is at its maximum. This expectation is borne out by Fig. 2e, which shows that $V_{W}/V_{W}^{\left(gen\right)}$ is indeed minimized close to $Nm_{\mathrm{crit}}$. Further, $V_{W}/V_{W}^{\left(gen\right)}$ is close to the Bulmer prediction (equation (14)), especially upon accounting for the effects of LD via effective parameters (solid lines).

In contrast to the variance within demes, genetic variance across the population as a whole can be much lower than the genic variance, i.e. $V_{T}/V_{T}^{\left(gen\right)}\ll 1$, especially at low levels of migration (Fig. 2d). This follows from equation (21): even when LD within demes is negligible, so that $V_{W}∼V_{W}^{\left(gen\right)}$, we might still have $V_{T}/V_{T}^{\left(gen\right)}\ll 1$, provided $F_{ST}$ at trait loci is high and $Q_{ST}$ low. This, in turn, requires that trait means be much less differentiated across demes than the individual loci that contribute to trait variation. This is precisely what occurs at low migration rates—different demes are nearly fixed for alternative alleles at any locus, resulting in high levels of polymorphism across the population as a whole, as well as high $F_{ST}$ (Fig. 2c). However, this does *not* mean that all possible genotypes (that can be constructed by randomly assorting the different alleles) are common in the population. Rather, any deme only supports specific nonrandom combinations of trait-increasing and trait-decreasing alleles with trait values very close to the trait optimum. This results in strongly negative LD between trait loci at the level of the population as a whole, which constrains trait values and trait means (Fig. 2f). Thus, at low migration rates, we have: $Q_{ST}\ll F_{ST}∼1$ and $V_{T}/V_{T}^{\left(gen\right)}\ll 1$.

We also obtain simple predictions for $Q_{ST}$ (equation (20)), which match simulations very well (Fig. 2f). As expected, $Q_{ST}$ is markedly lower than the neutral $F_{ST}$ (dotted black line)—a well-known consequence of spatially uniform stabilizing selection (Leinonen et al. 2013). Another way of seeing this is that $V_{T}∼V_{W}$ (Supplementary Fig. S6), meaning that the total genetic variance across the full population is largely due to variance within demes. Interestingly, while higher values of *Ns* lead to lower $Q_{ST}$ under very weak migration (Nm≪1), the effect of selection becomes weaker with increasing migration. This follows from the fact that for Nm≳1, the variance within demes is $V_{W}≃V_{W}^{\left(gen\right)}≃V_{*}$, so that:


(23) $$Q_{ST}\approx \frac{1}{1+4Nm+16N\mu L}$$

regardless of the strength of stabilizing selection. Thus, $Q_{ST}$ is significantly reduced relative to neutral $F_{ST}$ only if 2NμL, the average number of new trait-affecting mutations per deme per generation, is comparable to *Nm*, the number of migrants per deme per generation.

### Variance contributions of trait loci with different effect sizes

So far, we have considered traits influenced by loci of equal effect. To evaluate the robustness of our approximations, we now investigate alternative trait architectures. First, consider a simple example where 60%, 30%, and 10% of trait loci have weak $\left(N\frac{a^{2}}{2V_{s}}=Ns=0.25\right)$, moderate (Ns=1), and large (Ns=4) effects respectively. Figure 3a,b shows the proportion of genic variance – across the full population and within demes—contributed by each of the 3 types of loci, as a function of *Nm*.

Under very weak migration, $\bar{\;p}\;\bar{q}=1/4$ at any locus, regardless of its selection coefficient. Thus, the contribution of any locus *i* to total genic variance $V_{T}^{\left(gen\right)}$ scales with $Ns_{i}$, meaning that loci of large effect (Ns=4; purple) make disproportionately large contributions to $V_{T}^{\left(gen\right)}$ at low migration rates. With increasing migration, the contribution of a given type of locus drops sharply at the associated $Nm_{\mathrm{crit}}$ threshold (which is lower for larger *Ns*), leading to a compensatory increase in the *relative* contributions of the other types. As migration increases even further, the relative contribution of each type of locus to $V_{T}^{\left(gen\right)}$ becomes equal to the proportion of loci of that type (indicated by dashed horizontal lines). This is because for very large *Nm*, the structured population behaves as a large panmictic population of size $N_{\mathrm{tot}}=ND$, in which the average variance associated with any locus is the same (equal to $v_{*}=4\mu V_{s}$ under LE) for any locus with sufficiently large scaled effect size (i.e. $N_{\mathrm{tot}}s\gg 1$).

The effects of migration on the contributions of different loci to the within-deme genic variance $V_{W}^{\left(gen\right)}$ are broadly similar (Fig. 3b). The only difference is that large-effect loci do not contribute as disproportionately to $V_{W}^{\left(gen\right)}$ (as they do to $V_{T}^{\left(gen\right)}$) at low migration rates. This reflects the fact that per-locus contributions to $V_{W}^{\left(gen\right)}$ are relatively similar for loci of different effect size (see, e.g. Fig. 2b).

We next consider a more realistic trait architecture, where effect sizes are drawn from an exponential distribution with mean (scaled) effect $N\bar{s}=2$. Figure 3c,d shows the average genic variance contributed by each locus, to the full population and within demes, as a function of its scaled selective effect *Ns*. All variance contributions are scaled by $v_{*}=4\mu V_{s}$, the deterministic per-locus variance. As before, we see a good match between simulation results (crosses) and single-locus predictions (dashed lines), with the match improving if we use effective parameters to account for LD (solid lines).

To understand how population structure affects the relative contributions of small vs. large-effect loci to trait variance, first consider the Nm=0 limit (dark blue; Fig. 3d). As in the previous example, in this limit, $\bar{\;p}\;\bar{q}=1/4$ at any trait locus, regardless of its effect size. Thus, loci contribute to the total genic variance (across the full population) in proportion to their effect size (dark blue; Fig. 3c). By contrast, the average contribution of a trait locus to the within-deme variance is a nonmonotonic function of its effect size: it increases with effect size for low values of *Ns*, peaks at Ns∼1, and then declines again as *Ns* increases further, reaching its asymptotic value of $v_{*}=4\mu V_{s}$ for Ns≫1. Thus, with Nm=0, the largest contributions to within-deme trait variance are from loci of intermediate effect (see also Simons et al. 2018).

Weak migration between demes (Nm=0.1; light blue) broadens the allele frequency distribution within each deme, thus increasing the contributions of all trait loci to the within-deme variance, regardless of their effect size. At the same time, it depresses the contributions of large-effect loci to the total genic variance. This is because even weak migration is sufficient to drive the same allele to high frequency across the majority of demes at large-effect (strongly selected) loci, thus decreasing the overall level of polymorphism at such loci (Fig. 1b).

At even higher levels of migration (Nm=0.5 and 2; pale and dark red), variance contributions of loci with large to moderate effect tend to level out, approaching the deterministic prediction $v_{*}=4\mu V_{s}$, while loci of small effect (Ns≲0.12 for Nm=0.5, and Ns≲0.06 for Nm=2.0) contribute significantly less than $v_{*}$. At even larger migration rates, we expect the subdivided population to behave as a panmictic population of size $N_{\mathrm{tot}}=ND$, in which any locus with $N_{\mathrm{tot}}s≳1$ contributes $∼v_{*}$ to within-deme and total genic variance. In fact, the red curves in Fig. 3c,d (corresponding to Nm=2) are already very close to this panmictic expectation (dotted black curves), suggesting that even moderate population structure has little to no effect on the *expected* variance contributions of trait loci.

In addition to measuring per-locus variance contributions, we also track $F_{ST}$ at trait loci as a function of *Ns* (Fig. 3e). As in the case of a single underdominant locus (Fig. 1g), $F_{ST}$ at trait loci increases with their effect size only in highly structured populations (Nm=0 and 0.1 in Fig. 3e). Even modest levels of gene flow (Nm≳0.25) are enough to reverse this pattern, causing $F_{ST}$ to be lower for loci of larger effect.

### Distribution of variances across trait loci

The previous sections focused on the *expected* variance contributions of trait loci. However, the variance *v* contributed by any locus will fluctuate stochastically about this expected value over time and also across demes (in the case of within-deme variance). Thus, we can study the probability *distribution*  P(v) of *v* or, alternatively, the cumulative probability $F(v)=\int_{y=v}^{v_{\max}}P(y)\text{d}y$, where $v_{\max}$ is the maximum possible variance (corresponding to p=1/2). For a locus with selective effect *s* which is polymorphic in a given deme, the within-deme variance *v* can range from $v_{\min}=\frac{1}{2N}\left(1-\frac{1}{2N}\right)\frac{s}{\mu }$ to $v_{\max}=\left(\frac{1}{4}\right)\frac{s}{\mu }$ (in units of $v_{*}$).

We now investigate the behavior of F(v), with the aim of characterizing “outliers,” i.e. loci with unusually large contributions to the within-deme genic variance $V_{W}^{\left(gen\right)}$. We are interested in outliers because methods for identifying trait loci, e.g. GWAS, are more likely to detect loci with variance above a certain threshold (which decreases with increasing study size; Simons et al. 2018; Spence et al. 2026). Figure 4a shows the cumulative distribution F(v) of variance contributions of all trait loci, for a trait influenced by L=200 equal-effect loci with scaled effect size *Ns*, for 3 different values of *Ns* (colors) and Nm=2. As before, all variances are scaled by the deterministic expectation, $v_{*}=4\mu V_{s}$. Note that $v_{\min}$, the minimum possible variance associated with a polymorphic locus, is higher for loci of larger effect, while $F(v_{\min})$, the probability that the locus is polymorphic, is correspondingly smaller. Thus, the curves start at different points along the *x* and *y* axes.

**Fig. 4.**
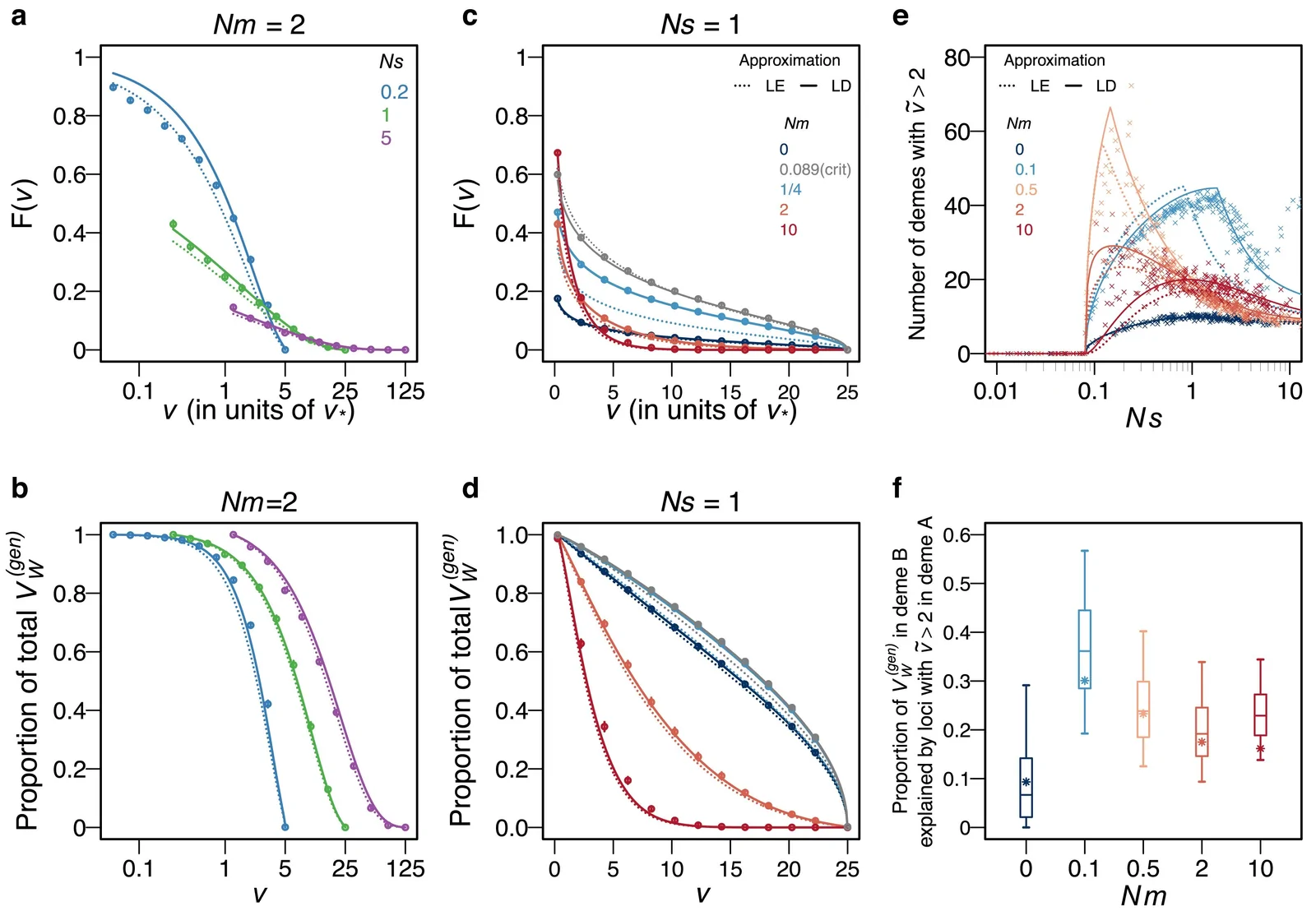
Distribution of the within-deme variance contributions of trait loci. a) and c) Cumulative probability F(v) that the variance contributed by a locus exceeds *v*, and b) and d) proportion of within-deme genic variance $V_{W}^{\left(gen\right)}$ explained by loci with variance exceeding *v*, as a function of *v*, for a trait influenced by L=200 loci of equal effect. Panels a) and b) show results for different values of *Ns*, the (scaled) locus effect size, for Nm=2. Panels c) and d) show results for different values of *Nm*, for Ns=1. All per-locus variances are measured in units of $v_{*}=4\mu V_{s}$, and can range from $v_{\min}=\frac{1}{2N}(1-\frac{1}{2N})\frac{s}{\mu }$ to $v_{\max}=(\frac{1}{4})\frac{s}{\mu }$ (in these units) for a locus with scaled effect *Ns*. Points show results of individual-based simulations with N=200, D=100, and Nμ=0.01; solid and dashed lines show LE and LD predictions, respectively. Panels e)–f) show results for a trait influenced by L=200 loci with exponentially distributed effect sizes with mean (scaled) effect $N\bar{s}=2$ (same distribution and simulations as in Fig. 3c–e). e) Expected number of demes in which a locus of effect size *Ns* makes a variance contribution exceeding $2v_{*}$, as a function of *Ns*, for different values of *Nm*. f) Proportion of genic variance in deme B explained by loci with variance contributions greater than $2v_{*}$ in deme A, for different values of *Nm*. Box plots show the 0.05, 0.25, 0.5, 0.75, and 0.95 quantiles of the distribution of the explained proportion of genic variance across different demes in individual-based simulations; the asterisks mark the theoretical LD prediction for the expected proportion.

A striking feature of this figure is that the distributions differ markedly across traits influenced by loci of small vs. large effect size, even though the mean of the distribution (the expected variance) is very similar, being $1.24v_{*}$, $1.19v_{*}$, and $1.09v_{*}$ for loci with Ns=0.2, 1, and 5, respectively in Fig. 4a. Broadly speaking, larger effect sizes result in a higher probability of more extreme, i.e. either zero or very large ($v/v_{*}\gg 1$), variance contributions. Conversely, loci with intermediate contributions (say, $1<v/v_{*}<5$) are associated with lower *Ns* values. For instance, in Fig. 4a, a locus with *Ns* equal to 0.2, 1 or 5 has a probability 0.45, 0.16, or 0.075 (computed as F(5)−F(1)) of making a contribution $1<v/v_{*}<5$ to $V_{W}^{\left(gen\right)}$.

One can further ask: what proportion of $V_{W}^{\left(gen\right)}$ is due to loci with unusually high variance contributions? In other words, when do a small number of such outliers account for most of the variance within demes? Figure 4b shows the proportion of within-deme genic variance explained by loci with contributions exceeding *v* as a function of *v*. Comparing the curves for the various *Ns* values, we see that outliers contribute more when effect sizes are larger (or equivalently, stabilizing selection is stronger). For instance, loci with variance exceeding $5v_{*}$ explain ∼83% of $V_{W}^{\left(gen\right)}$ if scaled effect sizes are Ns=5 (which corresponds to μ/s=0.002) and 60% of $V_{W}^{\left(gen\right)}$ if Ns=1 (i.e. μ/s=0.01).

We also explore how migration between demes affects the contribution of outlier loci to the within-deme trait variance for a trait with intermediate-effect loci (μ/s=0.01; Fig. 4c,d). Low levels of migration result in more outliers (as well as a higher proportion of variance explained by such outliers) compared to no migration, provided that $Nm<Nm_{\mathrm{crit}}$. However, a further increase in migration (beyond $Nm_{\mathrm{crit}}$) shifts F(v) toward lower values of *v*, thus decreasing the contribution of outliers to the within-deme variance. Nevertheless, even for migration as high as Nm=10 (dark red curve in Fig. 4c), a significant fraction (∼17%) of loci have variance contributions exceeding $2v_{*}$ within demes.

Finally, we also investigate the distribution of per-locus contributions to the total genic variance $V_{T}^{\left(gen\right)}$ (Supplementary Fig. S3). As expected, this distribution is quite narrow and concentrated about the expected value $\frac{Ns}{N\mu }\bar{\;p}\;\bar{q}$ in a population of large total size $N_{tot}=ND$. More precisely, the width of the distribution depends on the variance of $\bar{\;p}$, which approaches zero as $N_{tot}\to \infty$.

What implications do these results have for mapping the genetic basis of trait variation in structured populations? Returning to the example of trait loci with exponentially distributed effect sizes (Fig. 3c–e), we ask: in how many (out of the 100) demes, does a given locus make an atypically large variance contribution (say, $>2v_{*}$)? Figure 4e shows that loci with large contributions to multiple demes tend to be associated with intermediate *Ns* values. If we chose an even larger threshold (e.g. $5v_{*}$), then the typical *Ns* values associated with outliers would be higher, and any locus would be an outlier across fewer demes. However, regardless of the exact threshold for designating outliers, the probability that a locus is an outlier (possibly across multiple demes) is quite sensitive to *Ns*, even for *Nm* as large as 2 in Fig. 4e. This contrasts with the average variance per locus, which is largely independent of *Ns* for Ns≳0.1 and Nm>1 (Fig. 3d). Thus, even moderate to weak population structure can significantly influence the effect size distribution of detected QTL.

We also ask: what proportion of the genic variance in deme *B* is explained by loci identified as outliers (those with variance contributions $>2v_{*}$) in another deme A (Fig. 4f)? Interestingly, outliers in A explain the most variance in B at intermediate levels of migration. This is consistent with Fig. 4d, where we see that the relative contribution of outliers to genic variance is maximized close to the associated $Nm_{\mathrm{crit}}$ threshold. This means that trait variation is *less* polygenic, i.e. is explained by fewer loci with larger individual contributions, if $Nm∼Nm_{\mathrm{crit}}$. This causes the genetic basis of variation to also become more similar across different demes.

## Discussion

This paper investigates how genetic variation is maintained in a polygenic trait under stabilizing selection in a subdivided population. We show that the diffusion approximation for allele frequencies, coupled with basic quantitative genetic theory, can accurately predict the combined effects of selection and structure on QG variation, at least under a simple island model of population structure. Moreover, the effects of LD can be absorbed by effective migration and selection coefficients, allowing for a succinct description.

### How much (more) variation in structured populations?

We find that strong population structure can significantly increase diversity at trait loci across the population as a whole by allowing different alleles to persist at high frequency in different demes (Fig. 2a). However, since alleles only occur in specific multi-locus combinations (with trait values close to the optimum), trait variance remains quite constrained and is only modestly increased at intermediate levels of migration (Supplementary Fig. S6). Thus, under spatially uniform stabilizing selection, (strong) population structure is mainly a source of cryptic variation that may be “unlocked,” e.g. via experimental crosses between individuals from different subpopulations.

These conclusions are in line with those of other models with two demes (Phillips 1996; Lythgoe 1997; McDonald and Yeaman 2018) or of continuous space (Lande 1991; Goldstein and Holsinger 1992). In addition, our analysis furnishes an analytical prediction for the critical migration threshold, $Nm_{\mathrm{crit}}$, below which trait loci exhibit bistability and high levels of polymorphism. Under the island model, we find: $Nm_{\mathrm{crit}}\approx \frac{\sqrt{\left(\mu /s{)}^{2}+2(\mu /s\right)}+3(\mu /s)}{2(1-4(\mu /s))}$ for Ns≲1 and 2(μ/s)<1/2, where 2(μ/s) is also the expected heterozygosity at a trait locus in a panmictic population. Thus, in a scenario where typical heterozygosity per trait locus is (say) ≲0.1 in a panmictic population, *Nm* between demes must be at most ∼0.3 for population structure to significantly increase trait variation. Thus, broadly speaking, the rather weak levels of population structure found in most natural populations (see, e.g. Morjan and Rieseberg 2004) cannot significantly inflate QG variation, at least under spatially uniform stabilizing selection and with island-model migration (i.e. no isolation by distance).

There are two caveats to this. First, we focus on populations at mutation-selection-migration-drift *equilibrium*, which may not be attainable over biologically relevant timescales. For instance, imagine a scenario where a set of long-isolated populations (each with its own set of trait alleles) come into contact. While equilibrium predictions suggest that the same allele should eventually spread across all subpopulations at any locus of sufficiently large effect, this requires populations to cross a “fitness valley” (arising from selection against rare alleles at any locus), which can take very long under weak migration (see also Supplementary Fig. S5). Thus, structured populations may maintain much more variation than predicted by equilibrium theory.

Second, even though the *expected* variance contributed by (most) trait loci hardly changes beyond Nm∼2 (see, e.g. Figs. 2b and 3d), the *distribution* of variance contributions is sensitive to much higher levels of migration (Fig. 4c,d). For instance, in Fig. 4d, we see that the proportion of $V_{W}^{\left(gen\right)}$ explained by loci with atypically large contributions is very different for Nm=2 vs Nm=10. This further supports the idea that spatially restricted sampling of individuals (e.g. from a local subpopulation that is embedded in a larger population) may give a rather different picture of trait architecture compared to more widespread sampling (see also Battey et al. 2020; Steiner et al. 2025), even without local adaptation and under rather weak population structure.

### How much differentiation between subpopulations?

We analyze differentiation between subpopulations in two ways. We first ask whether the same (or alternative) alleles occur at high frequency in different demes. We then consider $F_{ST}$ and $Q_{ST}$, which quantify the relative contribution of among-population differences to genic diversity (at trait loci) and trait variance respectively.

In general, we expect different sets of alleles to be nearly fixed in populations with a long history of complete isolation (Nm∼0). This process of allelic differentiation between populations is actually accelerated by stabilizing selection, which increases the rate at which minor alleles are lost (Yair and Coop 2022). As discussed above, migration between populations alters this expectation, causing the same allele to reach high frequency across all demes, with the critical migration threshold, $Nm_{crit}$, at which this occurs being lower for loci of larger effect. A second observation is that while $F_{ST}$ at trait loci is always higher than the neutral expectation under weak migration, this pattern is actually reversed at higher levels of migration (Fig. 3e). These findings bear upon recent studies that aim to distinguish between alternative modes of selection (purifying vs. stabilizing vs. none) based on expected allele sharing at putative QTL between humans of different ancestries (Patel et al. 2024). These studies neglect gene flow between ancestries, which is known to have occurred (see, for instance Jouganous et al. 2017). This may bias conclusions, since, as we show here, even moderate levels of gene flow can affect allele sharing (e.g. as measured by $F_{ST}$) at selected loci (see, e.g. Fig. 3e). Moreover, since sites assayed in GWAS are often not causal themselves (but only in imperfect LD with the causal locus), gene flow may also affect the strength of LD between the assayed site and the causal locus it tags, thus further complicating interpretation.

Our results are also relevant to recent work exploring whether introgression between populations adapted to the same trait optimum may be impeded by stabilizing selection in the recipient population (Ragsdale 2025; Veller and Simons 2026). Such selection disfavors descendants of migrant individuals, that are *more* phenotypically variable than residents, provided $V_{B}^{\left(gen\right)}$ is high (see also equation (16)). However, as we show here, even low levels of migration are enough to drastically reduce among-population differentiation. Thus, spatially uniform stabilizing selection is unlikely to generate a significant barrier to gene flow unless populations have a long history of nearly complete isolation.

### Do the same or different alleles underlie trait variation in different subpopulations?

We find that an allele is more likely to be significantly associated with trait variation across different subpopulations when migration between populations is moderate (Fig. 4e,f). This is because very low or very high migration between demes drives alleles toward local or global fixation, respectively. This shifts the distribution of per-locus variance contributions to lower values, making it less likely that a locus will contribute significantly to multiple demes. This contradicts the general expectation that higher migration between populations should necessarily lead to more shared standing variation, thereby increasing parallel adaptation, e.g. in response to changing environments. Here, we demonstrate that while high migration necessarily increases allele sharing (by causing the same alleles to nearly fix) across sub-populations, it actually decreases shared polymorphism and standing variation (Fig. 4f).

What does this mean for mapping the genetic basis of variation in extended populations? Our results suggest that spatially limited sampling of individuals may actually be more useful (than broad sampling) for identifying trait loci. This is because the typical variance contributions of trait loci tend to be higher within individual subpopulations than across the population as a whole, especially for loci of large effect (contrast, e.g. Fig. 4a,b with Supplementary Fig. S3). This phenomenon is well known in the context of GWAS, where alleles significantly associated with trait variation in one population are usually not globally significant. This limits the “portability” of so-called polygenic scores (and other methods of genomic prediction) across populations (Duncan et al. 2019; Wang et al. 2020). As found by Yair and Coop (2022), stabilizing selection further reduces portability by reducing shared standing variation. However, here we show that this effect is quite sensitive to migration between populations (Fig. 4f), with intermediate levels of gene flow significantly mitigating the effects of stabilizing selection on shared variation.

### Toward a more quantitative interpretation of $Q_{ST}$ vs $F_{ST}$

In agreement with previous work (reviewed in Leinonen et al. 2013), we find that spatially uniform stabilizing selection reduces variation in trait means across subpopulations, causing $Q_{ST}$ to be lower than neutral $F_{ST}$ (Fig. 2f). In addition, we obtain a simple analytical prediction under the island model: $Q_{ST}=1/(1+4Nm+4N\frac{V_{W}}{V_{s}})$ (see equation (20)). Here, $V_{W}/V_{s}$ approaches 4μL (which is twice the total mutation rate for the trait) with increasing migration. This suggests that the difference between $Q_{ST}$ and neutral $F_{ST}$ should be pronounced when 4μL≫m, i.e. if population structure is strong and/or selected traits have large mutation targets. Moreover, this difference should be largely independent of both the strength of stabilizing selection and the effect sizes of trait loci (provided that *Nm* is not too low).

Thus, in theory, comparing $1/Q_{ST}$ with $1/F_{ST}$ (at neutral loci) can furnish an estimate of 4NμL, the total mutational input for the selected trait, or at least, provide a means of comparing mutation rates across traits. However, obtaining reliable estimates of $Q_{ST}$ is often difficult in practice (Whitlock 2008). Also, genetic correlations between traits would likely alter our simple prediction for $Q_{ST}$ for the focal trait, which will now be influenced by (spatially uniform or divergent) selection on other correlated traits. Thus, extending the model to multiple traits is a fruitful direction for future work that could more broadly clarify the extent to which $Q_{ST}$-$F_{ST}$ comparisons can provide information about (multivariate) selection in structured populations.

As noted by Le Corre and Kremer (2003), comparing $Q_{ST}$ to the average $F_{ST}$ across quantitative trait loci (which they refer to as $F_{STQ}$) also gives information about the proportion of trait variance that is due to LD between trait loci (see also our equation (21)). In particular, we find that even when LD within demes is negligible, there can be substantial negative LD in the population as a whole, provided that $F_{ST}$ at trait loci is high and $Q_{ST}$ low, which, in turn, requires Nm≲1 (Fig. 2d). Thus, while stabilizing selection only contributes weakly to LD in panmictic populations (unless trait loci are tightly linked), the combination of stabilizing selection and strong population structure can generate strong negative LD, causing genetic variance across the whole population to be up to an order of magnitude lower than genic variance (Fig. 2d).

### Extensions and open questions

We consider a simple model of selection in which fitness depends on a single polygenic trait under spatially uniform stabilizing selection. However, in reality, genetic variants may affect multiple traits, some of which may be subject to spatially uniform and others to divergent selection. Such a scenario, while highly relevant to evolutionary inference, remains under-explored in theoretical work (though see Guillaume and Whitlock 2007; MacPherson et al. 2015). In particular, we lack analytical predictions for how selection on multiple correlated traits and gene flow between populations together shape genetic variances and covariances across traits and loci. The diffusion approximation can be readily extended to allow for a more general relationship between the selection coefficient at a given locus and its effect on a focal trait (see, e.g. Simons et al. 2018). Thus, the theory developed here provides a natural starting point for investigating migration-selection equilibrium across many traits.

We also make various simplifying assumptions about trait architecture, namely that trait loci are unlinked, have additive effects (no phenotypic dominance) and that there is no mutation bias (so that expected trait means coincide with the trait optimum). The last assumption is crucial, since biased mutation (say, toward trait-increasing alleles) would cause the trait mean to deviate from the trait optimum, thus generating directional selection (in addition to underdominance) at each locus. Both directional selection and mutation bias can increase the frequency of alleles with, say, positive (compared to negative) effect on trait value (Charlesworth 2013): thus, it is unclear if their signatures can be distinguished in genomic data (Koch et al. 2024; Berg et al. 2025). Our analytical mean-field approach based on the diffusion approximation can potentially be extended to explore this question.

Finally, we consider a very simple island model of population structure, which has the advantage of being analytically tractable, thus allowing for detailed prediction. Such many-deme models represent at least as (un-)realistic a reference point for natural populations as two-deme models, which underlie the bulk of theory on selection in structured populations. However, both many and two-deme models fail to capture the complexity of selection across continuous space, wherein allele frequencies change over a characteristic spatial scale determined by the balance between dispersal and selection per locus (Barton 1999). For weakly selected loci, this spatial scale may be comparable to that associated with neutral isolation-by-distance (Nagylaki 1978), making it difficult to distinguish selected loci from the neutral background. Understanding to what extent signatures of (spatially uniform vs. heterogeneous) selection can be disentangled from those of spatial population structure in genomic data thus remains an open question, in sore need of more theoretical work.

## Supplementary Material

iyag185_Supplementary_Data

iyag185_Peer_Review_History

## Data Availability

Mathematica notebook with all numerical computations, Python code for individual-based simulation and R code for visualization are available at https://github.com/lijuan2010big/PopulationStructureAndStablizingSelection-Equilibrium.git. Details of various mathematical approximations and additional figures exploring the effects of varying mutation rates, number of loci and number of demes can be found in the Supplementary information. Supplemental material available at GENETICS online.
