# Supplementary material for: Effect of population structure and stabilizing selection on quantitative genetic variation
Source: Genetics. 2026 Jul 21;234(2):iyag185. doi: 10.1093/genetics/iyag185 (PMC13643806; doi:10.1093/genetics/iyag185)
Supplement: Supplementary file 1 [file iyag185_peer_review_history.pdf]

GENETICS

Effect of population structure and stabilizing selection on quantitative genetic variation

Juan Li, Joachim Hermisson, and Himani Sachdeva

NOTE: The reviews and decision letters are unedited and appear as submitted by the reviewers.
In extremely rare instances and as determined by a Senior Editor or the EIC, portions of a review may be redacted. If a review is signed, the reviewer has agreed to no longer remain anonymous.
The review history appears in chronological order.

Review Timeline:

Submission Date:	2026-03-31
Editorial Decision:	2026-05-11
Resubmission Received:	2026-06-15
Accepted:	2026-06-22

May 10, 2026

GENETICS-2026-309303
Effect of population structure and stabilizing selection on quantitative genetic variation

Dear Dr. Sachdeva:

Two experts in the field have reviewed your manuscript, and I have read it as well. I am pleased to inform you that, with minor revisions, it is potentially suitable for publication in GENETICS. The reviewers have a few comments and concerns that need to be addressed in a revised manuscript. You can read their reviews at the end of this email.

As you will see, the reviewers (and I) greatly appreciated the clear and thoughtful paper, and their comments are mostly suggestions for some additional discussion. In particular, please clarify Reviewer 1's query about what is being averaged over in some of the plots. I am hoping that Reviewer 1's point about mutation rate is easily addressed by some additional discussion, but you will know that better than I. Finally, thank you for making the code available. However, I'd like to see a little additional documentation: I see reasonably clear python scripts, but not much indication of how to use these to produce the results in the paper.

We look forward to receiving your revised manuscript. Please let the editorial office know approximately how long you expect to need for revisions.

Upon resubmission, please include:
1. A clean version of your manuscript;
2. A marked version of your manuscript in which you highlight significant revisions carried out in response to the major points raised by the editor/reviewers (track changes is acceptable if preferred);
3. A detailed response to the editor's/reviewers' comments and to the concerns listed above. Please reference line numbers in this response to aid the editors.

Prior to uploading your revised manuscript please format it according to GENETICS style and ensure you have all required elements. Author Guidelines are at https://academic.oup.com/genetics/pages/author-guidelines#section-12. These guidelines have been updated with new requirements and your careful attention is required to avoid delays.

Follow this link to submit the revised manuscript: Link Not Available

Sincerely,

Peter Ralph
Associate Editor
GENETICS

Approved by:
David Begun
Senior Editor
GENETICS

Reviewer #1 :

This is an excellent manuscript and will make a solid contribution to the field. I was very interested to read it and found it well-written and clear. It solidifies the general understanding from previous theory that maintenance of variation within populations is only likely to be strongly affected by population structure across a narrow range of parameter space in a model of stabilizing selection, mutation, and drift, providing analytical understanding of previous simulation-based results. It also extends these results, showing how loci of different effect sizes contribute to variance within and among populations, and explores the variance around expectations and contribution of outliers to genetic variance. I was generally interested to see a relatively limited effect of LD on the results, and good agreement between the predictions accounting for LD and simulations. Besides my first broad comment below, I have mainly minor comments, and commend the authors on writing a paper that was enjoyable and thought-provoking to review.

Broad comments:

- My main concern is that I find it difficult to evaluate how much the results depend upon the high mutation rate, as simulations for the Figures set N*mu = 0.01 and N = 200, which would put per-locus mutation rate at 0.00005 (several orders of magnitude higher than typical per-base pair mutation rates). As a starting point, N*m_crit would be much lower (based on 1D and Eq. 22), suggesting that at least for the single locus model, most natural populations will find themselves well in the region of m > m_crit and will behave according to the single population/panmictic intuition for the expectation. This is easy to intuit, and so a change in mutation rate should have a relatively predictable effect on Figure 3A/B, by shifting the positions of the relevant thresholds. The bigger question is whether the results related to variance around the expectation (Figure 4) would differ under lower mutation? I can guess that the qualitative patterns may be relatively robust, but become noisier with lower mutation rates, so perhaps the results are more difficult to evaluate, but some exploration/discussion of this would help, even if confined to the supp mat. When quantitative results are noted, how much would these depend on mutation? (e.g. line 704, 712). Similarly, it bears discussing how the magnitude of increase in VW and VT relative to V* (e.g. Figure 2; line 531 where "five times" is cited) depends upon mutation rate, and that the assumed value here is quite large. But I'm a bit confused here, as line 515 implies that VT will become enormous as mutation becomes small.

- A general concern I have with theoretical predictions of the expectation for Fst, Vw, etc, is it can obscure interesting behaviour only apparent in the variance of dynamics around the expectation, so it is nice to see the exploration in Figure 4 and discussion on lines 771-779. In a similar vein, are the points plotted for each locus in Figure 3 showing the average across a large number of generations? I was surprised to see how little noise they exhibit, but couldn't find if they are single-generation census or averages. This is somewhat related to my question about mutation rates, as I would imagine these values would be more noisy at lower mutation rates.

- I was particularly interested to note the non-monotonic pattern of dependence between the shape of the distribution and Nm in Figure 4E/F and found the discussion on lines 810-820 to be quite interesting. A natural follow-up would be to re-examine the findings of Macpherson + Nuismer on parallel adaptation from standing variation (https://onlinelibrary.wiley.com/doi/full/10.1111/jeb.13006) in light of this, but this is beyond the scope here.

Minor comments:

- line 20: I think this is meaning that the similarity decreases for higher migration rates? (as that would be counter-intuitive). This could be read as the variation decreases for higher migration rates (which would be intuitive).

- Seeing as Figure 1 already has colours, it might be helfpul to make the triangles (Ns = 5) and dashed line the same colour, as that would show that this line is specific to the triangles (this is already clear from the caption, but it would make it visually clearer). Also, it would seem helpful to include the m_crit vertical lines to panel 1G to further illustrate the point on line 442.

- Line 408: seems worth pointing out that the inverted U-shape is also contingent upon weak selection (N*s = 0.1) and not observed with strong selection in panel B.

- Line 515: I think "u" in denominator should be "mu"?

- Line 541: it seems strange to use the inequality mu/s < 1/24, as I would have thought N*s was more important here?

Reviewer #2 :

In this paper Li, Hermisson, and Sachdeva consider the equilibrium genetics of an infinite island model with a trait experiencing stabilizing selection. They analyze a model assuming no LD between alleles, and extend their approach via a sort of self-consistency / mean field strategy to handle LD induced by population structure (akin to ancestry LD) and stabilizing selection (the Bulmer effect). This results in a set of "effective" parameters that scale the predictions of the no LD model. The authors compare their theory to simulations showing that it's accurate. They also make a number of interesting observations, e.g., that there is a critical migration rate where the results qualitatively change. Overall, I found the paper, clear, extremely interesting, and of use to the broader genetics (and GENETICS) audience. I provide a few minor comments below in the hopes that they are useful to the authors.

I prefer to sign my reviews,
Jeffrey P. Spence

Minor comments:

- As I'm sure the authors know, the text on lines 91 and 92 of the main text is subtly wrong. There is not actually a heterozygote disadvantage (and in fact heterzygotes could have have the highest mean fitness in some situations, I believe), but the dynamics just so happen to match those of heterozygote disadvantage. I would rephrase this to make this subtle point a bit more clear.

- Preceding equation 6 in the main text, it may be helpful to make clear that this is in the absence of drift (or perhaps that it's the change in the expected allele frequency). This is made clear a few paragraphs later, but it was confusing to me on my first reading.

- Around line 305, an iterative strategy is described for determining the effective parameters. Is it obvious that there exists a solution to this system of equations, and that it's unique? Does the iterative approach converge to that solution? I don't think it's necessary to perform any analysis here, but it would be nice to at least include a statement about convergence in practice and dependence on the initializations of \{s_{i, eff}\} and m_{eff}.

- Around line 560 it would also be good to note that it likely violates the Normality assumption. This is even explicitly addressed in the appendix, and so including a pointer to that appendix here would be nice.

- On lines 628-631, I was a bit confused. Is this assuming that the effect size is large enough? For loci where a variant has an infinitesimally small effect, it should be effectively neutral, and its contribution to variance should scale quadratically in the effect size, no? Apologies if I am missing something here.

- On lines 682-684 it would be good to make clear that this threshold is with respect to the _expected_ GWAS signal. The actual association statistics in GWAS are random and so there is some variance around the expectation. I doubt that it's the best description of this distinction, but we tried to cover this in Appendix B of this paper: https://www.nature.com/articles/s41586-025-09703-7

- On line 734-735 it's stated that the genetic basis of variation becomes more similar across different demes when the migration rate is close to the critical value. What notion of similarity is being used here? When the migration rate is infinite, the allele frequencies should be the same across all demes, so it's hard for me to imagine that not being the most similar the genetic bases of variation could be across demes.

- In the appendix around line 166, it might be worth noting that assuming s \ll 1 is also required to obtain the diffusion approximation.

Typos:

- Line 732: "variance is maximum close" --> "variance is maximized close"

- Equation S.10 uses "Log" and equation S.11 uses \log, and the main text uses \ln. It would be good to make these all consistent.

- In Equation S.10 is \Gamma' the derivative of the gamma function with respect to its argument?

Associate Editor Comments:

GENETICS-2026-309303
Effect of population structure and stabilizing selection on quantitative genetic variation

Dear Dr. Sachdeva:

Two experts in the field have reviewed your manuscript, and I have read it as well. I am pleased to inform you that, with minor revisions, it is potentially suitable for publication in GENETICS. The reviewers have a few comments and concerns that need to be addressed in a revised manuscript. You can read their reviews at the end of this email.

As you will see, the reviewers (and I) greatly appreciated the clear and thoughtful paper, and their comments are mostly suggestions for some additional discussion. In particular, please clarify Reviewer 1's query about what is being averaged over in some of the plots. I am hoping that Reviewer 1's point about mutation rate is easily addressed by some additional discussion, but you will know that better than I. Finally, thank you for making the code available. However, I'd like to see a little additional documentation: I see reasonably clear python scripts, but not much indication of how to use these to produce the results in the paper.

We look forward to receiving your revised manuscript. Please let the editorial office know approximately how long you expect to need for revisions.

Upon resubmission, please include: 1. A clean version of your manuscript; 2. A marked version of your manuscript in which you highlight significant revisions carried out in response to the major points raised by the editor/reviewers (track changes is acceptable if preferred); 3. A detailed response to the editor's/reviewers' comments and to the concerns listed above. Please reference line numbers in this response to aid the editors.

Prior to uploading your revised manuscript please format it according to GENETICS style and ensure you have all required elements. Author Guidelines are at `https://academic.oup.com/genetics/pages/author-guidelines#section-12`. These guidelines have been updated with new requirements and your careful attention is required to avoid delays.

Sincerely,

Peter Ralph Associate Editor GENETICS

Approved by: David Begun Senior Editor GENETICS

We thank the reviewers for their suggestions and comments and respond to each of them below. We have also added more documentation to the code.

Reviewer #1 :

This is an excellent manuscript and will make a solid contribution to the field. I was very interested to read it and found it well-written and clear. It solidifies the general understanding from previous theory that maintenance of variation within populations is only likely to be strongly affected by population structure across a narrow range of parameter space in a model of stabilizing selection, mutation, and drift, providing analytical understanding of previous simulation-based results. It also extends these results, showing how loci of different effect sizes contribute to variance within and among populations, and explores the variance around expectations and contribution of outliers to genetic variance. I was generally interested to see a relatively limited effect of LD on the results, and good agreement between the predictions accounting for LD and simulations. Besides my first broad comment below, I have mainly minor comments, and commend the authors on writing a paper that was enjoyable and thought-provoking to review.

Broad comments:

- My main concern is that I find it difficult to evaluate how much the results depend upon the high mutation rate, as simulations for the Figures set N*mu = 0.01 and N = 200, which would put per-locus mutation rate at 0.00005 (several orders of magnitude higher than typical per-base pair mutation rates).
Mutation rates of $\sim 10^{-5}$ per locus are typical in quantitative and population genetic models (see e.g., the book by Reinhard Bürger), since loci usually refer to genes (i.e., some 1000 bases) rather than single base-pairs. Further, in the diffusion limit, only scaled parameters $N\mu$, Ns and Nm matter. Thus, as is often done in theory papers, we make use of this scaling property by choosing rather small population sizes N to reduce run-times in our individual-based simulations.

Crucially, as we discuss below, our results depend on $N\mu$ primarily via the ratio $N\mu/Ns = \mu/s$, if Ns is not too large (and neglecting the effects of LD, which are minor). With our choice of parameters, the ratio μ/s ranges between 0.001 and 0.1, which is a biologically meaningful range.

We have now made some changes to the text to highlight the dependence on μ/s (see details below). We have also added figures with different mutation rates in the SI.

As a starting point, Nm_{crit} would be much lower (based on 1D and Eq. 22), suggesting that at least for the single locus model, most natural populations will find themselves well in the region of $m > m_{crit}$ and will behave according to the single population/panmictic intuition for the expectation. This is easy to intuit, and so a change in mutation rate should have a relatively predictable effect on Figure 3A/B, by shifting the positions of the relevant thresholds.
As shown in Fig 1D and equation 22, for $Ns \lesssim 1$, the critical migration threshold Nm_{crit} depends on mutation rate primarily via μ/s, where $2\mu/s$ is also the heterozygosity at the trait locus in a large, panmictic population. Thus, we believe that it is more natural to think of Nm_{crit} as a function of μ/s rather than μ and s separately.This is also

highlighted in lines 767-771 (in the revised version) of the Discussion.

The bigger question is whether the results related to variance around the expectation (Figure 4) would differ under lower mutation? I can guess that the qualitative patterns may be relatively robust, but become noisier with lower mutation rates, so perhaps the results are more difficult to evaluate, but some exploration/discussion of this would help, even if confined to the supp mat. When quantitative results are noted, how much would these depend on mutation? (e.g. line 704, 712). Similarly, it bears discussing how the magnitude of increase in VW and VT relative to V* (e.g. Figure 2; line 531 where "five times" is cited) depends upon mutation rate, and that the assumed value here is quite large. But I'm a bit confused here, as line 515 implies that VT will become enormous as mutation becomes small.
We have now added a new section SI.f to the SI that shows that V_W and V_T relative to V_* (as shown in Fig. 3A, 3B), as well as the distribution of per-locus variance contributions (Figs. 4C, 4D), depend primarily on μ/s (and not μ and s separately). We also emphasise this in lines 505-507 of the manuscript. Also, when discussing quantitative results, e.g., on lines 541, 716-717, and 719 of revised version, we now cite the value of μ/s (rather than Ns), since this is the main determinant of various quantities.

- A general concern I have with theoretical predictions of the expectation for Fst, Vw, etc, is it can obscure interesting behaviour only apparent in the variance of dynamics around the expectation, so it is nice to see the exploration in Figure 4 and discussion on lines 771-779. In a similar vein, are the points plotted for each locus in Figure 3 showing the average across a large number of generations? I was surprised to see how little noise they exhibit, but couldn't find if they are single-generation census or averages. This is somewhat related to my question about mutation rates, as I would imagine these values would be more noisy at lower mutation rates.
Yes, as in the other figures, the simulation points in Figure 3 are obtained by averaging over generations and replicates, which is now clarified in the revised version. We also agree with the reviewer that there is much more information in the distribution of variances (as shown in Figure 4) – we address the issue of mutation rates and distribution of variances in our response to the previous comment.

- I was particularly interested to note the non-monotonic pattern of dependence between the shape of the distribution and Nm in Figure 4E/F and found the discussion on lines 810-820 to be quite interesting. A natural follow-up would be to re-examine the findings of Macpherson + Nuismer on parallel adaptation from standing variation (https://onlinelibrary.wiley.com/doi/full/10.1111/jeb.13006) in light of this, but this is beyond the scope here.
Yes, we agree that it would indeed be interesting and a natural next step to analyse how gene flow between populations influences parallel adaptation. We now also cite MacPherson and Nuismer on line 56, along with the other papers that consider parallel adaptation.

Minor comments:
- line 20: I think this is meaning that the similarity decreases for higher migration rates?

(as that would be counter-intuitive). This could be read as the variation decreases for higher migration rates (which would be intuitive).
We have now revised the sentence to make it clear.

- Seeing as Figure 1 already has colours, it might be helpful to make the triangles (Ns = 5) and dashed line the same colour, as that would show that this line is specific to the triangles (this is already clear from the caption, but it would make it visually clearer). Also, it would seem helpful to include the m_{crit} vertical lines to panel 1G to further illustrate the point on line 442.
We have updated Figure 1.

- Line 408: seems worth pointing out that the inverted U-shape is also contingent upon weak selection (N*s = 0.1) and not observed with strong selection in panel B.
Weak selection is implicit in the condition $Nm_{crit} > 1/2$, but we now also explicitly state that the inverted U-shape is observed if selection is weak.

- Line 515: I think "u" in denominator should be "mu"?
We have corrected this.

- Line 541: it seems strange to use the inequality $\mu/s < 1/24$, as I would have thought N*s was more important here?
The behaviour of FST that we describe basically requires $Nm_{crit} < 1/4$. Since Nm_{crit} depends very weakly on Ns and is largely determined by μ/s (figure 1g and eq. 22), the condition $Nm_{crit} < 1/4$ translates into $\mu/s \lesssim 1/24$. We have revised this to clarify the link with Nm_{crit}.

Reviewer #2 :
In this paper Li, Hermisson, and Sachdeva consider the equilibrium genetics of an infinite island model with a trait experiencing stabilizing selection. They analyze a model assuming no LD between alleles, and extend their approach via a sort of self-consistency / mean field strategy to handle LD induced by population structure (akin to ancestry LD) and stabilizing selection (the Bulmer effect). This results in a set of "effective" parameters that scale the predictions of the no LD model. The authors compare their theory to simulations showing that it's accurate. They also make a number of interesting observations, e.g., that there is a critical migration rate where the results qualitatively change. Overall, I found the paper, clear, extremely interesting, and of use to the broader genetics (and GENETICS) audience. I provide a few minor comments below in the hopes that they are useful to the authors.

I prefer to sign my reviews, Jeffrey P. Spence

Minor comments:

- As I'm sure the authors know, the text on lines 91 and 92 of the main text is subtly wrong. There is not actually a heterozygote disadvantage (and in fact heterozygotes could have the highest mean fitness in some situations, I believe), but the dynamics just so happen to match those of heterozygote disadvantage. I would rephrase this to make this subtle point a bit more clear.

We have rephrased the sentence to make it accurate.

- Preceding equation 6 in the main text, it may be helpful to make clear that this is in the absence of drift (or perhaps that it's the change in the expected allele frequency). This is made clear a few paragraphs later, but it was confusing to me on my first reading. We have now made explicit that this equation neglects drift.

- Around line 305, an iterative strategy is described for determining the effective parameters. Is it obvious that there exists a solution to this system of equations, and that it's unique? Does the iterative approach converge to that solution? I don't think it's necessary to perform any analysis here, but it would be nice to at least include a statement about convergence in practice and dependence on the initializations of $s_{i,eff}$ and m_{eff}. Given the complexity of the equations, it would be difficult to establish mathematically whether or not there is a unique solution. However, in practice, we observe that the iterations always converge to the same solution, regardless of how $\overline{p}$ (mean allele frequency in the metapopulation) is initialised. We have now added a sentence to this effect (lines 308-310). Note that we have also revised the description of the iterative procedure in the text, since this was not entirely accurate before.

- Around line 560 it would also be good to note that it likely violates the Normality assumption. This is even explicitly addressed in the appendix, and so including a pointer to that appendix here would be nice.
We now also note here that the assumption of normally distributed trait values is likely to be violated.

- On lines 628-631, I was a bit confused. Is this assuming that the effect size is large enough? For loci where a variant has an infinitesimally small effect, it should be effectively neutral, and its contribution to variance should scale quadratically in the effect size, no? Apologies if I am missing something here.
Yes, indeed, this is assuming that $N_{tot}s$ is large enough. We have now modified the sentence to make this clear.

- On lines 682-684 it would be good to make clear that this threshold is with respect to the expected GWAS signal. The actual association statistics in GWAS are random and so there is some variance around the expectation. I doubt that it's the best description of this distinction, but we tried to cover this in Appendix B of this paper: https://www.nature.com/articles/s41586-025-09703-7

We have now amended this sentence to reflect that GWAS is statistically more likely to detect loci with variance above a certain threshold.

- On line 734-735 it's stated that the genetic basis of variation becomes more similar across different demes when the migration rate is close to the critical value. What notion of similarity is being used here? When the migration rate is infinite, the allele frequencies should be the same across all demes, so it's hard for me to imagine that not being the most similar the genetic bases of variation could be across demes.
This sentence is part of a paragraph that describes Figure 4f, which shows the fraction

of variance in deme B that is explained by "outliers" (loci with above-average variance contributions) in deme A. This is the measure of similarity that is maximised at intermediate (as opposed to high) migration rates (Figures 4e and 4f). This somewhat counter-intuitive result is explained by the fact that for a locus to contribute significantly to trait variation across multiple demes, it must harbour an intermediate frequency polymorphism in multiple demes. However, as Nm increases, the minor allele frequency in the population as a whole decreases (see, e.g., Figure 1a). Thus, the probability (let's call it f) of observing an intermediate-frequency polymorphism at a given locus within any deme decreases, since any deme can be considered as a random subsample of the whole population (at least in the large Nm limit). Further, the probability of observing an intermediate-frequency polymorphism at a given locus across *multiple* demes becomes even lower (being $\sim f^2$ in the limit of many demes). In short, when considering loci that contribute significantly to trait variation, we are conditioning on an atypical event (the occurrence of an intermediate-frequency polymorphism in a deme) which become more atypical as Nm becomes large. We have also discussed this point in lines 823-832.

- In the appendix around line 166, it might be worth noting that assuming $s \ll 1$ is also required to obtain the diffusion approximation.
We have now added this point.

Typos:
- Line 732: "variance is maximum close" → "variance is maximized close"
Modified.
- Equation S.10 uses "Log" and equation S.11 uses log, and the main text uses ln. It would be good to make these all consistent.
We consistently use log now.
- In Equation S.10 is Γ' the derivative of the gamma function with respect to its argument?
We now clarify this in the text.

June 22, 2026
RE: GENETICS-2026-309620

Dr. Himani Sachdeva
University of Vienna
Faculty of Mathematics
Oskar-Morgenstern Platz 1
Vienna
Austria

Dear Dr. Sachdeva:

Congratulations, your manuscript titled "Effect of population structure and stabilizing selection on quantitative genetic variation" is accepted for publication in GENETICS! Many thanks for submitting your research to the journal.

I appreciate the good work you've put into these revisions, and have no further suggestions. Before publication, however, I would like to request that you provide some description of how to use the code that is provided in the github repository - probably, write in the README what needs to be done. (Filenames are fairly self-explanatory, but nonetheless, something should be written.)

To Proceed to Publication:
1. Format your article according to GENETICS style: https://academic.oup.com/genetics/pages/author-guidelines

2. Ensure that you comply with data and community resource citation guidelines:
https://academic.oup.com/genetics/pages/author-guidelines#section-5-9-2

3. Upload your final files at https://genetics.msubmit.net

4. Add oupsupport@scipris.com and genetics.oup@novatechset.com (or the domains @scipris.com and @novatechset.com) to your email program's "safe senders" list. You will be contacted by both at various points during the production process.

Notes:
- Your currently-accepted manuscript (unedited, as submitted, reviewed, and accepted) will be published at GENETICS and deposited into PubMed as an Advance Access article. Notify sourcefiles@thegsajournals.org before signing your license if you do not wish to publish your article via Advance Access.

- We invite you to submit an original color figure related to your paper for consideration as cover art. Please note that submissions created with AI image generation tools are not eligible. Please email your submission to the editorial office or upload it with your final files. You can submit a small-sized image for evaluation, and if selected, the final image must be a TIFF file 2513px wide by 3263px high (8.375 by 10.875 inches; resolution of 600ppi). Please avoid graphs and small type.

- After files are sent to Oxford University Press we use SciPris to manage article licensing and payment. If you do not have a SciPris account, you will receive an email from no-reply@scipris.com to sign up to use Oxford University Press' author portal. After logging in, follow the online instructions to sign your license and arrange any payment due.

If you have any questions or encounter any problems while uploading your accepted manuscript files, please email the editorial office at sourcefiles@thegsajournals.org.

Sincerely,

Peter Ralph
Associate Editor
GENETICS

Approved by:
David Begun
Senior Editor
GENETICS
