# Supplementary material for: Effect of population structure and stabilizing selection on quantitative genetic variation
Source: Genetics. 2026 Jul 21;234(2):iyag185. doi: 10.1093/genetics/iyag185 (PMC13643806; doi:10.1093/genetics/iyag185)
Supplement: Supplementary file 2 [file iyag185_supplementary_data.pdf]

Effect of population structure and stabilizing selection on quantitative genetic variation

Juan Li, Joachim Hermisson and Himani Sachdeva

Supplementary information

SI.a Weak and strong selection approximations for a single underdominant locus.

In principle, the equilibrium allele frequency distribution in eq. (7) fully specifies the behaviour of a single underdominant locus at mutation-selection-migration-drift equilibrium under the island model. However, this distribution $\Phi[p|\overline{p}]$ is a function of $\overline{p}$, the mean allele frequency across the whole population, which is itself obtained by numerically integrating over $\Phi[p|\overline{p}]$, thus necessitating a numerical solution. In this Appendix, we derive simpler analytical expressions for various quantities by considering the weak selection and strong selection approximations of the full equilibrium distribution.

For clarity, we re-write equation (7) below, omitting the subscript i for the locus (since we are only considering a single locus) as well as the subscript j for the deme. Then we have:

$$\Phi[p|\overline{p}] \propto e^{-4Nspq}\ p^{4Nm\overline{p}+4N\mu-1}\ q^{4Nm\overline{q}+4N\mu-1} \tag{S.1}$$

where $\overline{p}$ can be found by numerically solving:

$$\overline{p} = \mathbb{E}[p|\overline{p}] = \frac{\int_0^1 dp\, p\, \Phi[p|\overline{p}]}{\int_0^1 dp\, \Phi[p|\overline{p}]} \tag{S.2}$$

Weak selection. For $Ns \ll 1$, we can approximate the allele frequency distribution (eq. (S.1)) as: $\Phi[p|\overline{p}] \approx p^{4Nm\overline{p}+4N\mu-1} q^{4Nm\overline{q}+4N\mu-1} (1 - 4Nspq)$. Substituting into eq. (S.2), and retaining only first-order terms in small parameters (i.e., in Ns and $N\mu$) yields a cubic equation for $\overline{p}$. This has the following three solutions:

$$\overline{p} = \frac{1}{2}, \frac{1}{2}\left(1 \pm \sqrt{1 - \frac{(1+2Nm)(1+4Nm)}{2(Nm)^2}\frac{\mu}{s}} \right) \tag{S.3}$$

By evaluating the stability of the three equilibria, we find that there is a critical migration threshold Nm_{crit}, such that $\overline{p} = 1/2$ is the only stable equilibrium for $Nm < Nm_{\text{crit}}$ and $\overline{p} = \frac{1}{2}\left(1 \pm \sqrt{1 - \frac{(1+2Nm)(1+4Nm)}{2(Nm)^2}\frac{\mu}{s}} \right)$ are the only stable equilibria for $Nm > Nm_{\text{crit}}$. The critical migration threshold (at which the stability of the equilibria changes) can be obtained as the Nm value for which the expression under the square root in eq. (S.3) becomes zero. This yields:

$$Nm_{\text{crit}} \approx \frac{\sqrt{(\mu/s)^2 + 2(\mu/s)} + 3(\mu/s)}{2(1 - 4(\mu/s))} \tag{S.4}$$

Note that for $\mu/s \ll 1$, eq. (S.4) reduces to $Nm_{\text{crit}} \approx \sqrt{\frac{\mu}{2s}}$, which is eq. 25b of Barton and Rouhani (1993). However, eq. (S.4) is more accurate for larger values of μ/s (in practice, for $\mu/s > 0.01$), and applies as long as $\mu/s < 1/4$ (which is the deterministic criterion for bistable allele frequencies at an underdominant locus in a panmictic population).

Equation (S.3) can also be used to calculate the critical selection strength Ns_{crit} (above which bistability occurs) as a function of Nm, at least in a parameter regime in which $Ns_{\text{crit}} \ll 1$ (which requires Nm to be reasonably large). This is simply given by:

$$Ns_{\text{crit}} = \frac{(1+2Nm)(1+4Nm)}{2(Nm)^2} N\mu \tag{S.5}$$

The above threshold diverges as $Nm \to 0$ (and is thus not accurate in the low-migration regime). At the other extreme of high migration, we have $Ns_{\text{crit}} \approx 4N\mu$, or $s_{\text{crit}} = 4\mu$, which is the critical selection threshold for bistability in a large panmictic population.

We can also calculate $\mathbb{E}[pq]$ by explicitly integrating over the weak selection approximation for $\Phi[p]$. Then, using the expression for $\overline{p}$ in eq. (S.3), and retaining terms up to first order in Ns and $N\mu$, gives the following expression for $F_{ST} = 1 - \mathbb{E}[pq]/(\overline{p}\,\overline{q})$:

$$F_{ST} \approx \begin{cases} \dfrac{1}{1+4Nm}\left(1 + \dfrac{8Nm\,Ns}{(1+4Nm)(3+4Nm)} - \dfrac{8N\mu}{(1+4Nm)}\right) & \text{for } Nm < Nm_{\text{crit}} \\ \dfrac{1}{1+4Nm}\left(1 + \dfrac{8Nm\,(1-4Nm)\,Ns}{(1+2Nm)(1+4Nm)(3+4Nm)} + \dfrac{64\,Nm\,N\mu}{(1+4Nm)(3+4Nm)}\right) & \text{for } Nm > Nm_{\text{crit}} \end{cases} \tag{S.6}$$

Equation (S.6) shows that for $Nm > Nm_{\text{crit}}$ and in the limit $N\mu \to 0$, the first-order correction to F_{ST} due to selection changes sign precisely at $Nm = 1/4$. Thus, F_{ST} is greater than $1/(1+4Nm)$ (the neutral expectation) for $Nm < 1/4$, and less than $1/(1+4Nm)$ for $Nm > 1/4$, provided that $Nm_{\text{crit}} < 1/4$.

Strong selection. If selection is strong and migration sufficiently weak, then the equilibrium distribution is sharply concentrated near the boundaries $p = 0$ and $p = 1$. Then we can approximate the distribution near each boundary by a Gamma distribution, such that: $\Phi[p] = \Phi_0[p] \approx p^{4Nm\overline{p}+4N\mu-1}e^{-4Nsp}$ near $p = 0$, and $\Phi[p] = \Phi_1[p] \approx (1-p)^{4Nm(1-\overline{p})+4N\mu-1}e^{-4Ns(1-p)}$ near $p = 1$. We then use the fact that $\Phi_0(p)$ and $\Phi_1(p)$ fall off rapidly to zero on moving away from the boundaries $p = 0$ and $p = 1$ respectively. This allows us to approximate the expected allele frequency as:

$$\mathbb{E}[p] \approx \frac{\int_0^1 dp\, p\,(\Phi_0[p] + \Phi_1[p])}{\int_0^1 dp\,(\Phi_0[p] + \Phi_1[p])} \approx \frac{\int_0^\infty dp\, p\,\Phi_0[p] + \int_{-\infty}^1 dp\, p\,\Phi_1[p]}{\int_0^\infty dp\,\Phi_0[p] + \int_{-\infty}^1 dp\,\Phi_1[p]} \tag{S.7}$$

Similarly, the expected heterozygosity can be approximated as:

$$\mathbb{E}[pq] \approx \frac{\int_0^\infty dp\, pq\,\Phi_0[p] + \int_{-\infty}^1 dp\, pq\,\Phi_1[p]}{\int_0^\infty dp\,\Phi_0[p] + \int_{-\infty}^1 dp\,\Phi_1[p]} \tag{S.8}$$

Now using $E[p] = \overline{p}$ gives the following equation for $\overline{p}$:

$$\overline{p} = f(\overline{p}) = \frac{4(4Ns)^{4Nm(2\overline{p}-1)}(Ns - N\mu - Nm(1-\overline{p}))\Gamma[4(N\mu + Nm(1-\overline{p}))] + \Gamma[1 + 4N\mu + 4Nm\overline{p}]}{4Ns\left(\;(4Ns)^{4Nm(2\overline{p}-1)}\Gamma[4(Nm(1-\overline{p}) + N\mu)] + \Gamma[4(N\mu + Nm\overline{p})]\;\right)} \tag{S.9}$$

where $\Gamma[x]$ denotes the Gamma function. Equation (S.9) can be solved numerically to obtain the equilibrium allele frequency $\overline{p}$. As in the case of weak selection, multiple equilibria are possible, whose stability changes with increasing Nm. For $Nm < Nm_{\text{crit}}$, we have a single stable equilibrium at $\overline{p} = 1/2$, whereas for $Nm > Nm_{\text{crit}}$, there are two stable equilibria – one lower than and one greater than $1/2$. One can obtain the critical threshold Nm_{crit} by noting that it is that value of Nm at which $f'(1/2) = 1$. This gives:

$$Ns - Nm_{\text{crit}} + 2Nm_{\text{crit}}(Nm_{\text{crit}} - Ns + 2N\mu)\left(\log[4Ns] - \frac{\Gamma'[2Nm_{\text{crit}} + 4N\mu]}{\Gamma[2Nm_{\text{crit}} + 4N\mu]}\right) = 0 \quad \text{(S.10)}$$

where $\Gamma'[x]$ denotes the derivative of the Gamma function with respect to x. Equation (S.10) can be solved numerically to obtain the critical migration threshold Nm_{crit}. Alternatively, we can obtain an explicit closed-form expression in the limit where $N\mu \ll 1$ (which is a natural assumption) and Nm_{crit} is also sufficiently small (which requires Ns to be large). Then we have: $\frac{\Gamma'[4N\mu+2Nm_{\text{crit}}]}{\Gamma[4N\mu+2Nm_{\text{crit}}]} \approx -\left(\gamma + \frac{1}{4N\mu+2Nm_{\text{crit}}}\right)$. Substituting this into eq. (S.10) and retaining terms up to first order in the small parameters Nm_{crit} and $N\mu$ gives a quadratic equation for Nm_{crit}, which can be solved to obtain Nm_{crit} as a function of Ns and $N\mu$. To lowest order in $N\mu$, this is:

$$Nm_{\text{crit}} \approx \sqrt{\frac{N\mu}{\gamma + \log[4Ns]}} \quad \text{(S.11)}$$

This is the same as eq. 21b of Barton and Rouhani (1993) with an erroneous factor of 2 corrected.

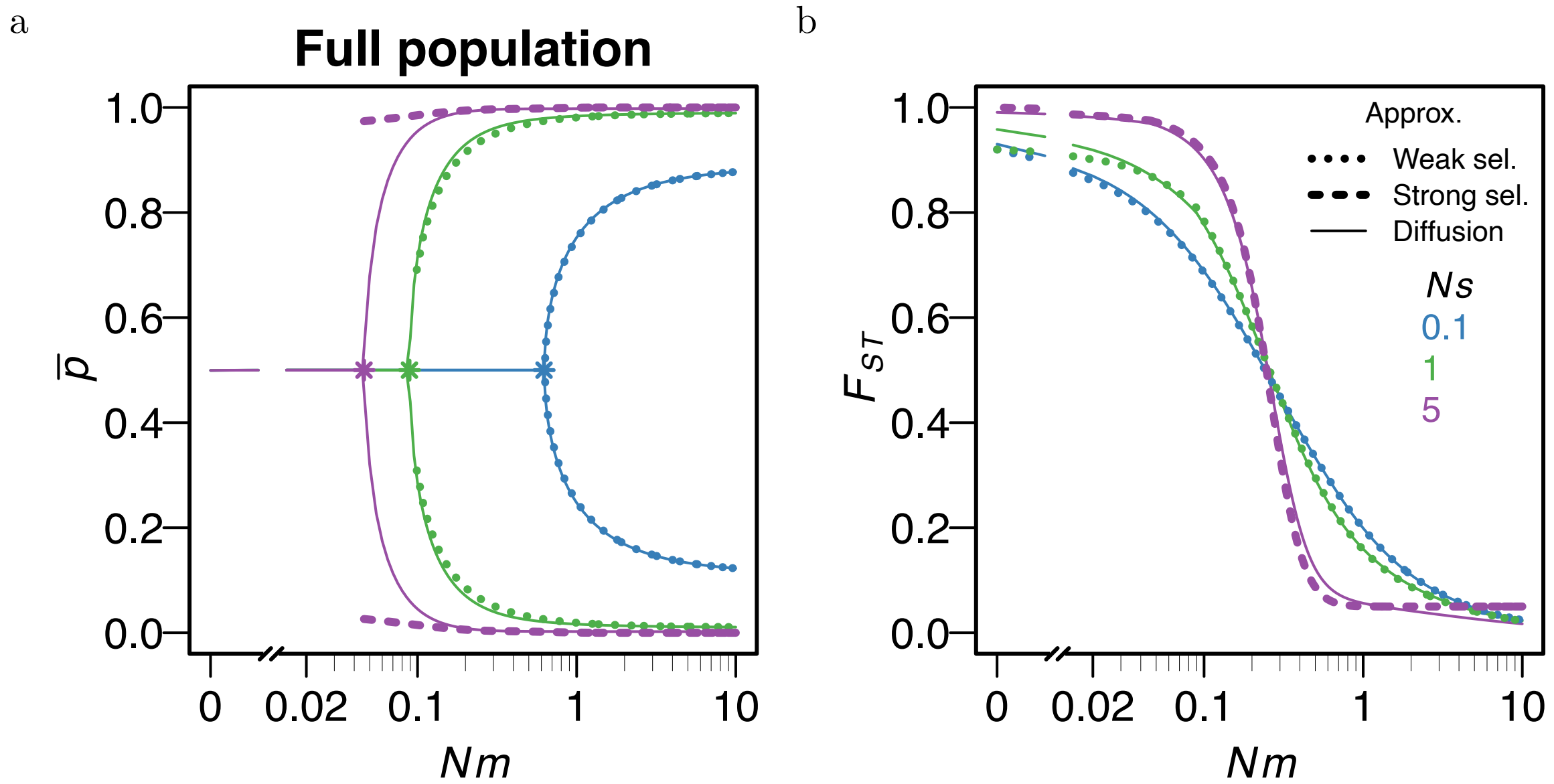

Figure S1. Comparison of the full diffusion approximation (solid lines) with the strong selection (dashed line) and weak selection (dotted line) approximations for a single underdominant locus. (a) Mean allele frequency across the full population $\overline{p}$ and (b) F_{ST} at the selected locus as a function of Nm for three different values of Ns, assuming $N\mu = 0.01$. Asterisks in (a) mark the critical migration rates (Nm_{crit}) as predicted by the diffusion approximation. Weak selection approximations are reasonably accurate for Ns as large as 1.

We can also obtain explicit expression for $\overline{p}$ and F_{ST} when Nm is sufficiently larger than Nm_{crit} that $\overline{p} \ll 1$, but small enough that the allele frequency distribution can be approximated

by the sum of Gamma distributions (as above). Then assuming that $\overline{p}$ is $\mathcal{O}(\mu/s)$, Taylor expanding equation (S.9) in powers of μ/s and solving the resultant equation gives:

$$\overline{p} \approx \frac{Ns}{Ns-Nm}\frac{(4Ns)^{4Nm}+4(Ns-Nm)\Gamma[4Nm]}{(4Ns)^{4Nm}-4Nm\Gamma[4Nm]}\left(\frac{\mu}{s}\right)+\mathcal{O}\left(\left(\frac{\mu}{s}\right)^2\right) \tag{S.12}$$

Similarly, we have:

$$\begin{aligned} F_{ST} &\approx \frac{1}{4Ns}\frac{(4Ns)^{4Nm}+4(Nm+4(Ns-Nm)^2)\Gamma[4Nm]}{(4Ns)^{4Nm}+4(Ns-Nm)\Gamma[4Nm]}+\mathcal{O}\left(\frac{\mu}{s}\right) \\ &= \frac{1}{2}+(\log(4\gamma Ns))\left(\frac{1}{4}-Nm\right)+\mathcal{O}\left(\frac{\mu}{s}\right)+\mathcal{O}\left(\frac{1}{Ns}\right)+\mathcal{O}\left(\left(\frac{1}{4}-Nm\right)^2\right) \end{aligned} \tag{S.13}$$

As in the case of weak selection, we see that F_{ST} exceeds the neutral expectation $1/(1+4Nm)$ for $Nm \lesssim 1/4$ but falls below it for $Nm \gtrsim 1/4$. This change in F_{ST} occurs at precisely $Nm = 1/4$ in the limit of very strong selection, i.e., as $\mu/s \to 0$ and $Ns \to \infty$.

We now test the weak selection and strong selection approximations by comparing $\overline{p}$ and F_{ST} as obtained numerically from equations (S.1) and (S.2) (i.e., the full diffusion approximation) against the predictions of equations (S.3) and (S.6) (for weak selection) and equations (S.12) and (S.13) (for strong selection). As evident from Figure S1, the weak selection predictions are very accurate for $Ns = 0.1$, and are reasonable even for $Ns = 1$, regardless of Nm. By contrast, the strong selection predictions for $\overline{p}$ are only accurate for $Nm \lesssim 1$: this is not surprising since the strong selection approximation assumes that allele frequency distribution are concentrated near $p = 0$ and $p = 1$ (which requires migration to be weak).

SI.b Effective migration rate at a trait locus.

In this section, we derive an expression for the effective migration rate m_{eff} at a site that is unlinked to any trait locus. This derivation follows the basic argument in Surendranadh and Sachdeva (2025), which we recapitulate here for completeness.

In general, $m_{\text{eff}} < m$ if immigrants and/or their descendants have lower fitness, on average, than the residents in any given deme (where 'residents' will be defined more precisely below). In this case, immigrant alleles are selected against, even if they are neutral, due to their association with less fit genetic backgrounds. However, as long as the focal allele is unlinked to any selected locus, such associations break down over $\lesssim 10$ generations – much faster than the timescales that govern single-locus dynamics (which depend on $1/m$, $1/s$, N, etc.). Thus, we can describe the effect of indirect selection arising from these transient associations simply by substituting an effective migration rate (instead of the raw migration rate) into the single-locus equations for allele frequency dynamics (Sachdeva, 2022).

In the limit $m \to 0$, the ratio m_{eff}/m is simply equal to the average reproductive value of migrants (Kobayashi et al., 2008), which is their long-term contribution to the gene pool of the recipient population, relative to that of residents. Alternatively, m_{eff}/m can be thought of as the factor by which the fixation probability of a neutral migrant allele is reduced relative to that of a neutral resident allele. Further progress can be made by noting that under random mating, the probability of mating between individuals with *recent* migrant ancestry is $\mathcal{O}(m^2)$, and thus, can be neglected for $m \ll 1$. Thus, in this limit, migrant individuals mate primarily with residents to produce $F1$ hybrids, which mate primarily with residents to produce first-generation backcrosses and so on.

In the following, we first derive an expression for the effective rate of migration from a specific donor deme to a specific recipient deme; we will then take the expectation over all pairs

of donor and recipient demes. We will use the subscript '0' to denote a migrant, '1' to denote the first-generation descendant of the migrant (an F1 individual), and $i = 2, 3, \ldots$ to denote the ith generation descendant of the migrant (an $(i-1)^{th}$ generation backcross). Further, we will designate any individual within the recipient deme with no migrant ancestor in the last g generations as a 'resident' (represented by the subscript 'r'). The cutoff g is arbitrary and will later be assumed to approach infinity. Let $\overline{W}_r$ and $\overline{W}_i$ denote the mean fitness among residents and among i^{th} generation descendants of migrants. Then, following Westram et al. (2022), we can express the effective migration rate at an unlinked locus as:

$$m_{\text{eff}}/m = \lim_{g\to\infty} \prod_{i=0}^{g} \frac{\overline{W}_i}{\overline{W}_r} \tag{S.14}$$

To find the various $\overline{W}_i$, we further assume that trait values within any population subgroup, say the ith generation descendants of migrants, follow a distribution $P_i(z)$, which is approximately normal with mean $\overline{z}_i$ and variance V_i. Similarly, resident trait values are also approximately normally distributed with mean $\overline{z}_r$ and variance V_r. We can then express $\overline{W}_r$ and $\overline{W}_i$ as:

$$\overline{W}_i = \exp\left[-\frac{(\overline{z}_i - z_{\text{opt}})^2}{2V_s} - \frac{V_i}{2V_s}\right] \qquad \overline{W}_r = \exp\left[-\frac{(\overline{z}_r - z_{\text{opt}})^2}{2V_s} - \frac{V_r}{2V_s}\right] \tag{S.15}$$

In the next step, we establish how the mean and variance of trait values within any population subgroup are related to those in the parental subgroups. More specifically, one can express the trait value distribution $P_i(z)$ in the ith subgroup as:

$$P_i(z) \propto \int dx \int dy\, W(x)\, W(y)\, P_r(x)\, P_{i-1}(y) \left(\frac{1}{\sqrt{2\pi V_{r,i-1}^{(seg)}}} \exp\left[-\frac{(z-\frac{x+y}{2})^2}{2V_{r,i-1}^{(seg)}}\right]\right) \tag{S.16}$$

Here, x and y are respectively the trait values associated with the resident parent and the parent who is an $(i-1)^{th}$ generation descendant of a migrant; $P_r(x)$ and $P_{i-1}(y)$ are the corresponding trait value distributions. Parental trait value distributions are weighted by the fitness function $W(z) = e^{-\frac{(z-z_{\text{opt}})^2}{2V_s}}$. Finally, the last term describes the inheritance of a quantitative trait under the infinitesimal model: the trait value z of the offspring is assumed to be normally distributed around the average of the two parents with variance equal to the segregation variance $V_{r,i-1}^{(seg)}$ released by mating between residents and $(i-1)^{th}$ generation descendants of migrants.

Assuming, as before, that $P_r(x)$ and the various $P_i(y)$ are normal distributions, and integrating over x and y yields the following recursions:

$$\overline{z}_i = \left(\frac{V_s}{V_s + V_{i-1}}\right)\frac{\overline{z}_{i-1}}{2} + \left(\frac{V_s}{V_s + V_r}\right)\frac{\overline{z}_r}{2} + \frac{2V_{i-1}V_r + V_s(V_{i-1} + V_r)}{2(V_{i-1} + V_s)(V_r + V_s)} z_{\text{opt}} \tag{S.17a}$$

$$V_i = \frac{V_s(2V_{i-1}V_r + V_s(V_{i-1} + V_r))}{4(V_{i-1} + V_s)(V_r + V_s)} + V_{r,i-1}^{(seg)} \tag{S.17b}$$

The above equations simplify considerably if $V_r/V_s \ll 1$ and $V_i/V_s \ll 1$. Then, to zeroth order in V_r/V_s and V_i/V_s, we have:

$$\overline{z}_i \simeq \frac{\overline{z}_{i-1}}{2} + \frac{\overline{z}_r}{2} \qquad V_i \simeq \frac{V_r}{4} + \frac{V_{i-1}}{4} + V_{r,i-1}^{(seg)} \qquad \text{for } i \geq 1 \tag{S.18}$$

However, even without assuming variances to be small, as long as $\overline{z}_r = \overline{z}_0 = z_{\text{opt}}$, we have: $\overline{z}_i = z_{\text{opt}}$, i.e., all population subgroups (regardless of proportion of migrant ancestry) have mean trait value equal to the trait optimum.

In the last step, we establish how the various segregation variances $V_{r,i}^{(seg)}$ are related to the within-deme and among-deme genic variances. Let $p_{r,k}$ and $p_{i,k}$ denote respectively the allele frequency at trait locus k among residents and among i^{th} generation descendants of migrants. As above, if $V_r/V_s \ll 1$ and $V_i/V_s \ll 1$, then to leading order in these small terms, we have:

$$p_{i,k} \simeq \frac{p_{i-1,k}}{2} + \frac{p_{r,k}}{2} = \frac{1}{2^i} p_{0,k} + \left(1 - \frac{1}{2^i}\right) p_{r,k} \tag{S.19}$$

This is simply the change in allele frequencies due to segregation at each trait locus and neglecting the effects of selection.

We can now express the various segregation variances in terms of allele frequencies as:

$$V_{r,r}^{(seg)} = \sum_{k=1}^{L} \alpha_k^2 \, p_{r,k}(1 - p_{r,k}) \tag{S.20a}$$

$$V_{r,0}^{(seg)} = \sum_{k=1}^{L} \frac{\alpha_k^2}{2} \left[p_{r,k}(1 - p_{r,k}) + p_{0,k}(1 - p_{0,k})\right] \tag{S.20b}$$

$$\begin{aligned} V_{r,i}^{(seg)} &= \sum_{k=1}^{L} \frac{\alpha_k^2}{4} \left[\overbrace{2p_{r,k}(1-p_{r,k})}^{\substack{\text{heterozygosity at locus} \\ k \text{ in resident parent}}} + \overbrace{p_{r,k}(1-p_{i-1,k}) + p_{i-1,k}(1-p_{r,k})}^{\substack{\text{heterozygosity at locus} \\ k \text{ in } (i-1)^{th} \text{ gen. parent}}} \right] \qquad \text{for } i \geq 1 \\ &= \left(2 - \frac{1}{2^i}\right) \sum_{k=1}^{L} \frac{\alpha_k^2}{2} p_{r,k}(1-p_{r,k}) + \left(\frac{1}{2^i}\right) \sum_{k=1}^{L} \frac{\alpha_k^2}{2} p_{0,k}(1-p_{0,k}) + \left(\frac{1}{2^i}\right) \sum_{k=1}^{L} \frac{\alpha_k^2}{2} (p_{r,k} - p_{0,k})^2 \end{aligned} \tag{S.20c}$$

In expectation, we have: $\mathbb{E}[p_{r,k}(1-p_{r,k})] = \mathbb{E}[p_{0,k}(1-p_{0,k})]$ and $\mathbb{E}[(p_{r,k} - p_{0,k})^2] = \mathbb{E}[(p_{r,k} - \overline{p}_k)^2] + \mathbb{E}[(p_{0,k} - \overline{p}_k)^2] = 2\mathbb{E}[(p_{r,k} - \overline{p}_k)^2]$ (for the infinite-island model), where $\overline{p}_k$ is the mean allele frequency at locus k across the entire population. Thus, in expectation, we have:

$$V_{r,r}^{(seg)} = V_{r,0}^{(seg)} = \frac{V_r}{2} = \frac{V_W^{(gen)}}{2} \qquad V_{r,i}^{(seg)} = \frac{V_W^{(gen)}}{2} + \frac{V_B^{(gen)}}{2^{i+1}} \qquad \text{for } i \geq 1 \tag{S.21}$$

where $V_B^{(gen)} = V_T^{(gen)} - V_W^{(gen)}$ is the among-deme genic variance.

Combining equations (S.14) and (S.15) with the observation that $\overline{z}_i = \overline{z}_r = z_{\text{opt}}$ for all i (in expectation), we obtain:

$$m_{\text{eff}}/m = \exp\left[-\sum_{i=0}^{\infty} \left(\frac{V_i - V_r}{2V_s}\right)\right] \tag{S.22}$$

Combining equations (S.18) and (S.21) with the observation that $V_r = V_0$ gives:

$$\begin{aligned} \sum_{i=0}^{\infty} (V_i - V_r) = \sum_{i=1}^{\infty} (V_i - V_r) &= \frac{1}{4} \sum_{i=1}^{\infty} (V_{i-1} - V_r) + \sum_{i=1}^{\infty} \left(V_{r,i-1}^{(seg)} - \frac{V_r}{2}\right) \\ &= \frac{1}{4} \sum_{i=1}^{\infty} (V_{i-1} - V_r) + V_B^{(gen)} \sum_{i=2}^{\infty} \left(\frac{1}{2^i}\right) \\ \implies \sum_{i=0}^{\infty} (V_i - V_r) &= \left(\frac{4}{3}\right)\left(\frac{1}{2}\right) V_B^{(gen)} = \frac{2}{3} V_B^{(gen)} \end{aligned} \tag{S.23}$$

Thus, we finally have:

$$\begin{aligned} \frac{m_{\text{eff}}}{m} &= \exp\left[-\frac{1}{3}\left(\frac{V_B^{(gen)}}{V_s}\right)\right] \\ &\approx 1-\frac{1}{3}\left(\frac{V_B^{(gen)}}{V_s}\right) \quad \text{if} \quad V_B^{(gen)}/V_s \ll 1 \end{aligned} \tag{S.24}$$

This is the same as equation 16 of Veller and Simons (2024) (which is expressed in terms of F_{ST} at trait loci instead of $V_B^{(gen)}$) up to a factor of 2. The factor of 2 difference arises from the different (two-island vs. infinite-island) models analysed in the two studies.

The derivation above is strictly valid only for a neutral locus that is unlinked to trait loci. By contrast, m_{eff} at a trait locus should also depend on s, the strength of direct selection at that locus. However, this effect can be neglected if $s \ll 1$ (Sachdeva, 2022), allowing us to approximate m_{eff} at a trait locus via eq. (S.24). The assumption that $s \ll 1$ also underlies the diffusion approximation and is not overly restrictive as Ns can still be substantial.

SI.c Factors influencing the accuracy of theoretical approximations

Our theoretical approximations are based on the infinite-island ($D \to \infty$) assumption and also rely on the standard quantitative genetic assumption of normally distributed trait values (which is justified in the $L \to \infty$ limit). Here, we explore to what extent departures from these assumptions might cause discrepancies between simulations and theory.

Finite number of demes. Taking the $D \to \infty$ limit (i.e., assuming an infinite number of islands) allows us to treat $\overline{p}$, the mean allele frequency at any locus, as deterministic. However, in a finite population, $\overline{p}$ will fluctuate about its expected value, causing the total heterozygosity $H_T = \overline{p}(1-\overline{p})$ to be lower in expectation than its predicted value (since $\mathbb{E}[\overline{p}(1-\overline{p})] = \mathbb{E}[\overline{p}](1-\mathbb{E}[\overline{p}]) - \text{Var}[\overline{p}]$). This is indeed what we observe in Figs. 1e for a single underdominant locus under weak selection ($Ns = 0.1$) and for Nm less than or close to the predicted Nm_{crit}. Theory predicts $\overline{p} = 1/2$ for these parameters. However, in simulations, $\overline{p}$ varies considerably around this expected value, especially for $Ns < 1$ and with $D = 100$. This reduces H_T as well as H_W, since deviations of $\overline{p}$ from 1/2 also tend to reduce $\mathbb{E}[2pq]$. A similar pattern is also observed for the polygenic trait (Figs. 2a and 2b) for $Ns = 0.1$.

To investigate this further, we consider a specific parameter combination ($Ns = 0.1$, $N\mu = 0.01$ and $Nm = 0.5$) for which there is a significant discrepancy between simulations and theoretical predictions for a single underdominant locus in Figs. 1e and 1f. Varying the number of demes D while keeping all other parameters fixed (Fig. S2a), we find that H_T and H_W (as measured in simulations) both increase with D, approaching the infinite-island prediction as D becomes large. This is consistent with the fact that fluctuations in $\overline{p}$ (about the expected value of 1/2) fall with increasing D.

We then ask: is the variance of $\overline{p}$ influenced directly by the number of demes or only via its effect on the total population size $N_{tot} = ND$? To distinguish between these two possibilities, we perform simulations where D is increased, while simultaneously decreasing N, increasing m, and keeping μ and s fixed (Fig. S2b). Thus, $N_{\text{tot}}\mu = ND\mu$ (total mutation rate across the full population), Nm (number of migrants per deme) and μ/s (strength of mutation relative to selection) remain unchanged as the D increases. Figure S2b shows that there is very little effect of D on heterozygosities in this case. Further, the discrepancy between simulations and theory is lower for larger values of $N_{\text{tot}}\mu$ (circles vs. squares). This suggests that for a given value of Nm and μ/s, the number of demes affects heterozygosities only via $N_{\text{tot}}\mu$.

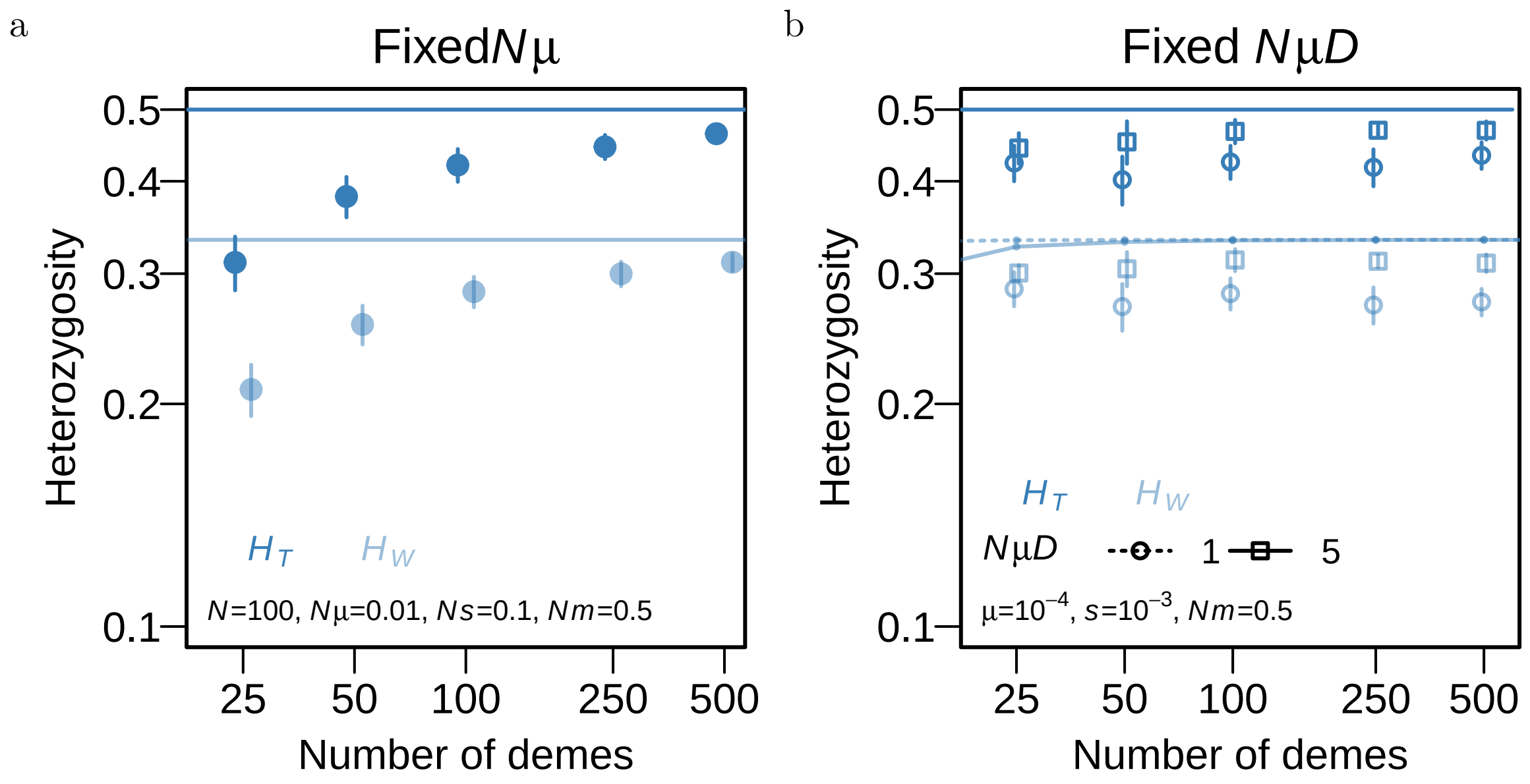

Figure S2. Within-deme heterozygosity (H_W; light blue) and total heterozygosity (H_T; dark blue) at a single underdominant locus as a function of number of demes D with (a) $N\mu = 0.01$ and $N = 100$ held constant (b) $N\mu D = 1$ (circles) or $N\mu D = 5$ (squares) constant. Symbols show the results of individual-based simulations while lines indicate theoretical predictions for the infinite-island model. In (b), the mutation rate and selection coefficient are fixed at $\mu = 10^{-4}$ and $s = 10^{-3}$; the number of demes D, the population size per deme N, and migration rate m are varied simultaneously such that Nm and $N\mu D$ remain constant. Simulation results approach the infinite-island prediction with increasing total population size $N_{\text{tot}} = ND$ (in both (a) and (b)).

In addition to tracking the expected H_T and H_W, we can also examine their distributions, or (in the case of the polygenic trait) the distribution of the variances contributed by individual trait loci to the full population or within demes. Figures 4a and 4c show that theory for the infinite-island model accurately predicts the distribution of per-locus contributions to $V_W^{(gen)}$ in simulations with $D = 100$ demes. However, infinite-island theory cannot predict the distribution of per-locus contributions to $V_T^{(gen)}$, since (by definition) in the $D \to \infty$ limit, the variance of $\overline{p}$ at any locus must be zero, and the distribution of any function of $\overline{p}$ must be a delta function. Thus, we investigate the effect of finite D on the distribution of per-locus contributions to $V_T^{(gen)}$ using only simulations. Figure S3 shows that, as expected, the distribution of variance contributions to $V_T^{(gen)}$ becomes narrower as the number of demes (and consequently, N_{tot}) increases. Also, the distribution of variance contributions to $V_T^{(gen)}$ is comparably broad across loci of different effect sizes. Finally, we note that per-locus contributions to $V_T^{(gen)}$ are much less broadly distributed than the corresponding contributions to $V_W^{(gen)}$ (compare Figs. 4a and 4c with Fig. S3 for $D = 100$). Thus, any locus is much more likely to contribute disproportionately to within-deme than total genic variance.

Finite number of trait loci. In the large L limit, the distribution of trait values is expected to be approximately normal. This assumption of normality underlies various QG predictions in the main text including eqs. (14), (15), (16) and (17). However, when selection per locus is strong, then the number of polymorphic loci can be rather small even if L is not too small. In

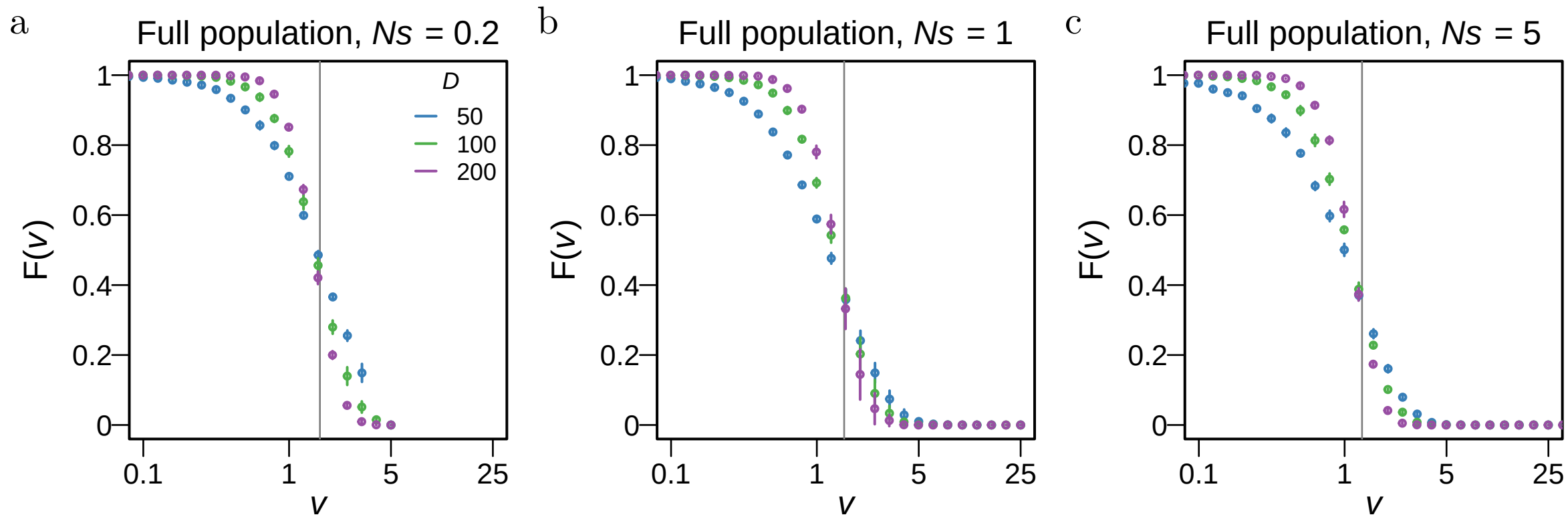

Figure S3. Cumulative probability $F(v)$ that the contribution of a locus to $V_T^{(gen)}$ exceeds v, as a function of v (which is measured in units of v_*), for a trait influenced by $L = 200$ loci of equal effect with scaled effect size per locus (a) $Ns = 0.1$ (b) $Ns = 1.0$ and (c) $Ns = 5$. Plots are obtained from individual-based simulations with different numbers of demes D (different colors), with other parameters being $N = 200$, $Nm = 2$ and $N\mu = 0.01$. The vertical line shows the expected variance contribution in the $D \to \infty$ limit of the model (as obtained from LD predictions). As D increases, the cumulative distribution falls off more and more sharply, and is expected to approach a step function in the limit of very large D.

this case, we expect the assumption of normally distributed trait values to break down, resulting in deviations between simulation results and theoretical predictions for the polygenic trait. This is also what we see in the Figure 2 for traits influenced by equal-effect loci of relatively large effect ($Ns = 5$). In these figures, simulation results deviate significantly from LD predictions for both low and high values of Nm (when overall levels of variation are low) and are, in fact, closer to the LE prediction.

To better understand how the number of loci, L, influences the match between simulations and theory, we focus on a simple scenario of stabilizing selection on a trait influenced by equal-effect loci in a *single* panmictic population. In this case, the LE prediction for the (scaled) genic variance depend only on two scaled parameters: $N\mu$ and Ns (and is independent of L), while LD predictions depend additionally on V_W^{gen}/V_s (which is $4\mu L$ times the scaled genic variance). This means that LD predictions for the (scaled) genic variance depend on three parameters: $N\mu$, Ns and μL. Thus, to test if simulation results approach LD predictions as L is increased, these three composite parameters must be held constant. This is accomplished by varying parameters according to: $L \to xL$, $N \to xN$, $\mu \to \mu/x$, and $V_s \to V_s x$ (where x is an arbitrary scaling factor), such that $N\mu$, $Ns = N\frac{\alpha^2}{2V_s}$ and μL remain unchanged. Note that under this kind of parameter scaling, increasing L causes $2N\mu L$ (which is the population-wide rate of trait-affecting mutations) to increase by the same factor.

Figure S4 shows how the scaled genic variance of a trait with $Ns = 5$, $N\mu = 0.01$ and $\mu L = 0.01$ depends on $N\mu L$. As in Fig. 2, genic variance in simulations is significantly lower than the LD prediction for small values of $N\mu L$. However, increasing $N\mu L$ (by increasing L using the parameter scaling described above) causes simulation results to converge towards the LD prediction.

SI.d The approach to equilibrium

In the main paper, we focus on equilibrium predictions for genic variance. However, populations may equilibrate very slowly under conditions of low gene flow, especially in scenarios where equi-

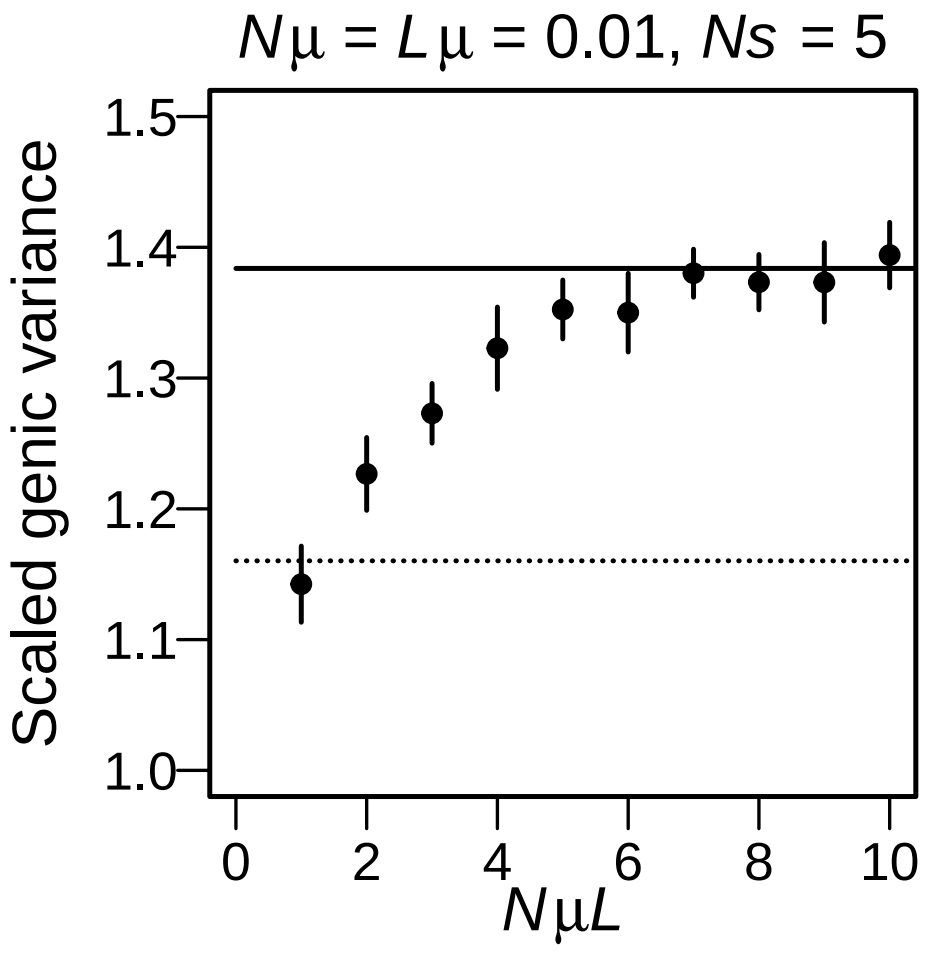

Figure S4. Scaled genic variance in a single panmictic population under stabilizing selection as a function of $N\mu L$, for $N\mu = 0.01$, $Ns = 5$ and $L\mu = 0.01$. Symbols show results of individual-based simulations; the dashed line shows the LE prediction; the solid line shows the prediction that accounts for the effects of LD on genic variance via s_{eff}. To vary $N\mu L$, we increase N, simultaneously decreasing μ and increasing L and V_s by the same factor, such that $N\mu$, $L\mu$ and $Ns = N\frac{\alpha^2}{2V_s}$ remain unchanged. Simulation results approach the LD prediction at higher values of $N\mu L$. However, for smaller $N\mu L$, there is a discrepancy between the two due to there being too few polymorphic trait loci.

libration involves the crossing of fitness valleys by individual demes (that may initially occupy different adaptive peaks). To get some intuition for how the initial distribution of genotypes in a subdivided population influences equilibration times, we simulate a single underdominant locus as well as the full polygenic trait starting with two contrasting initial conditions IC1 and IC2 (described below) and under different levels of migration (Fig. S5).

Under the first kind of initial condition (IC1), we start with all demes having the same allele frequency at a given locus, while under IC2, allele frequencies across different demes are sampled independently from a Beta distribution (details in caption of Fig. S5). In essence, all demes either initially occupy the same adaptive peak (IC1) or are uniformly distributed across all possible adaptive peaks (IC2). Thus, the total heterozygosity across the population as a whole is either extremely low (IC1) or at its maximum possible value of 1/2 (IC2).

As expected, equilibration is slower for smaller values of Nm (Fig. S5). Further, equilibration is also slower when it involves a qualitative change in the state of the population from its initial state. For example, equilibration takes longer when a population must evolve from initially high H_T (IC2; different alleles common in different demes) to low H_T (where the same allele is common in all demes) than when it starts already at low H_T: compare, for example, IC1 vs. IC2 for $Nm = 0.2$ in Figs. S5c and S5d.

SI.e Within-deme and total genetic variance

Figures 2e and 2d in the main paper show how the ratio of the genetic to genic variance changes with the strength of selection and migration. This ratio is less than 1 (especially for strong selection and weak migration), as there is negative LD between trait-increasing (or trait-decreasing) alleles within demes and at the level of the full population. Thus, $V_W/V_W^{(gen)}$ and $V_T/V_T^{(gen)}$ can be used as proxies for the total amount of LD between trait loci within demes and across the full population respectively.

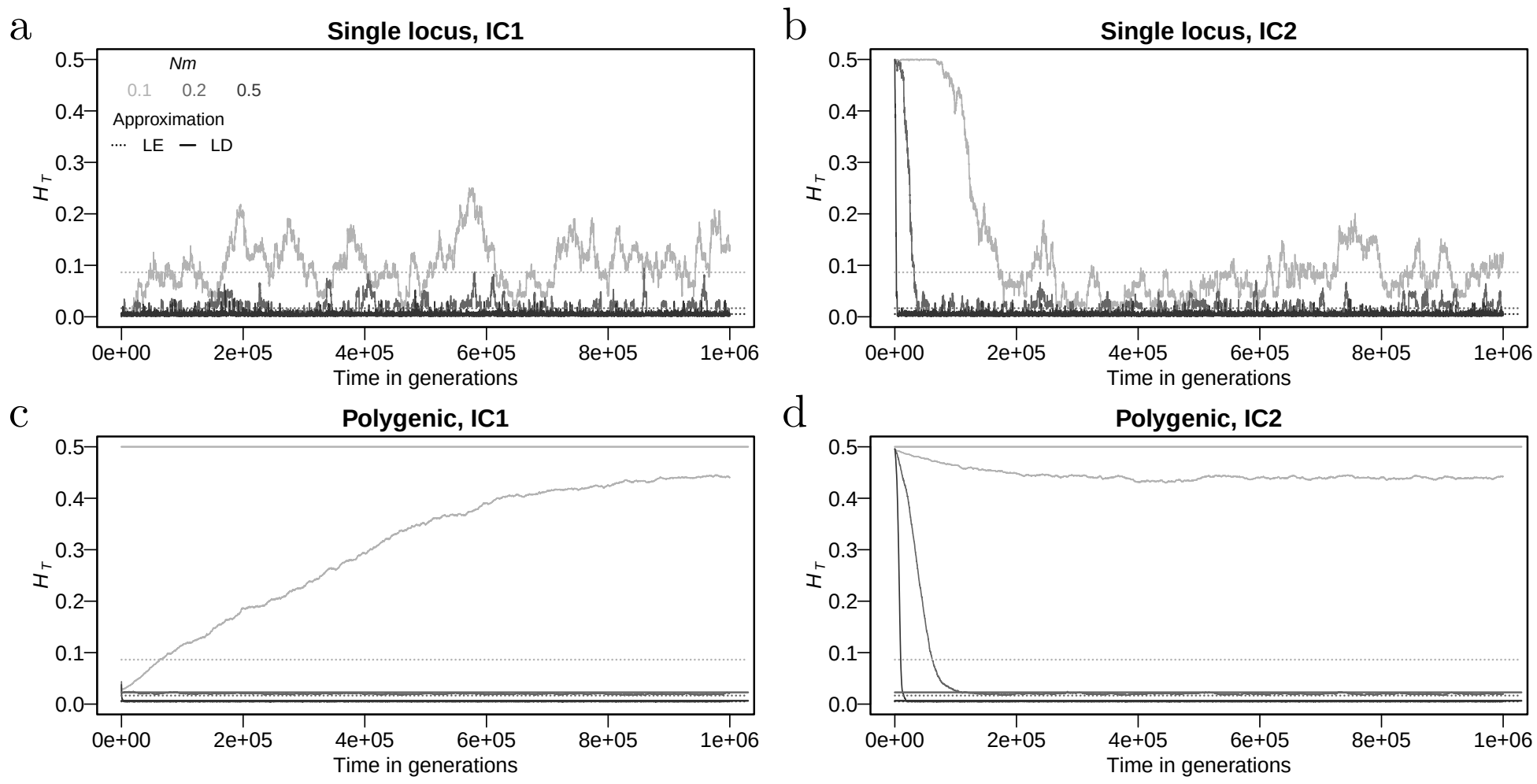

Figure S5. Total heterozygosity $H_T = 2\overline{p}\,\overline{q}$ as a function of time for: (a),(b) a single underdominant locus; (c),(d) $L = 200$ equal-effect loci influencing a trait under stabilizing selection (where heterozygosities are averaged across all loci), for three different values of Nm. Plots show results of a single simulation run with $D = 100, N = 200$, $N\mu = 0.01$ and $Ns = 5$, that are initialised in one of two ways. In plots (a) and (c), the mean allele frequency $\overline{p}$ at any locus is drawn from a Beta distribution $B[4N\mu, 4N\mu]$; the allele frequency in each deme is then set to this value (IC1). In plots (b) and (d), allele frequencies in each deme are drawn independently from a Beta distribution $B[4N\mu, 4N\mu]$ (IC2). The two initial conditions IC1 and IC2 thus correspond respectively to very low or maximal H_T at the start of the simulation. Dashed lines represent the theoretical predictions for H_T at equilibrium. Equilibration takes longer at lower levels of migration. Further, equilibration is slower when it involves a large change in H_T from very low to high values (or vice versa), as this necessitates the crossing of fitness valleys (generated by underdominant selection) at multiple loci.

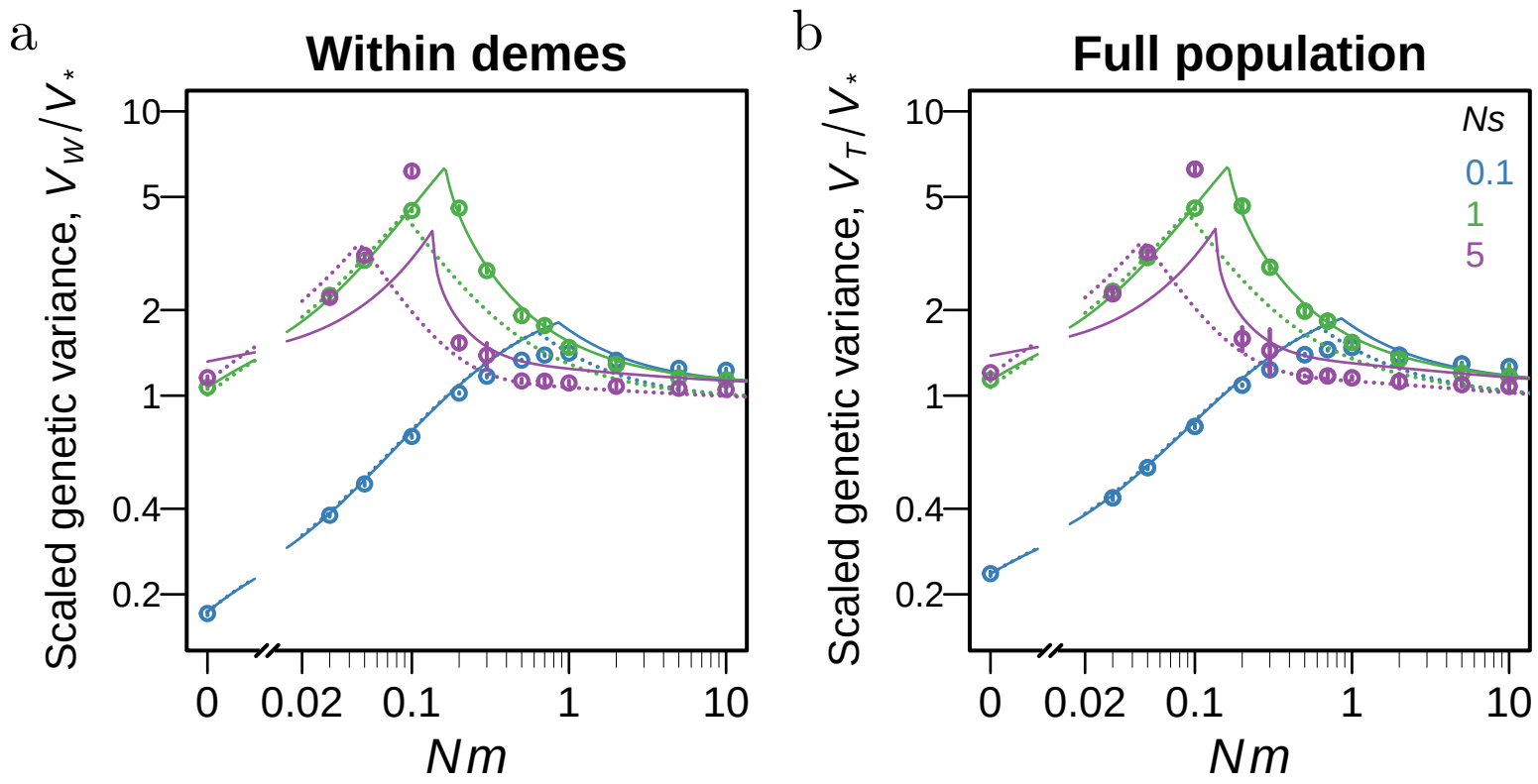

Figure S6. (a) Within-deme genetic variance V_W/V_* and (b) total genetic variance V_T/V_* as a function of Nm for a trait influenced by equal-effect loci, for 3 different values of Ns (scaled effect size per locus). All parameters are the same as in Fig. 2 ($D = 100$, $N = 200$, $L = 200$ and $N\mu = 0.01$), and symbols show results from the same set of individual-based simulations. Theoretical predictions are based on eqs. (14), (19) and (21), together with single-locus theory for the genic variances – either with or without effective parameters (solid vs. dashed lines).

Here, we examine how the genetic variances V_W and V_T themselves (rather than scaled by genic variance) depend on various parameters for a trait with equal-effect loci. Figure S6 shows that both the within-deme and total genetic variances are maximized at intermediate levels of migration ($Nm \simeq Nm_{\text{crit}}$). Further, while strong population structure inflates total genic variance by orders of magnitude (Fig. 2a), especially for traits influenced by many large-effect loci, its effect on the total genetic variance is quite modest. This is consistent with the observation that $V_T/V_T^{(gen)} \ll 1$ at low migration rates (Fig. 2d). Finally, we note that $V_T \simeq V_W$, except for the case of $Ns = 0.1$ (and $Nm \ll 1$). This is consistent with the rather low values of Q_{ST} observed in Fig. 2f and reflects the fact that very little of the total genetic variance in a population is due to among-deme differences in trait means, since these are quite tightly constrained under stabilizing selection.

SI.f Dependence of various quantities on mutation rate per locus.

In the main paper, we always keep the scaled mutation rate fixed at $N\mu = 0.01$. Here, we explore the dependence of various quantities on mutation rate based on our theoretical predictions. Figures S7a,b show that the scaled genic variances depend primarily on the ratio μ/s, and not on $N\mu$ and Ns separately. Similarly the distribution of per-locus contributions to $V_W^{(gen)}$ and the proportion of $V_W^{(gen)}$ accounted for by loci with variance exceeding a certain value (see also Figures 4a, 4b) also depend primarily on μ/s (Figures S7c,d).

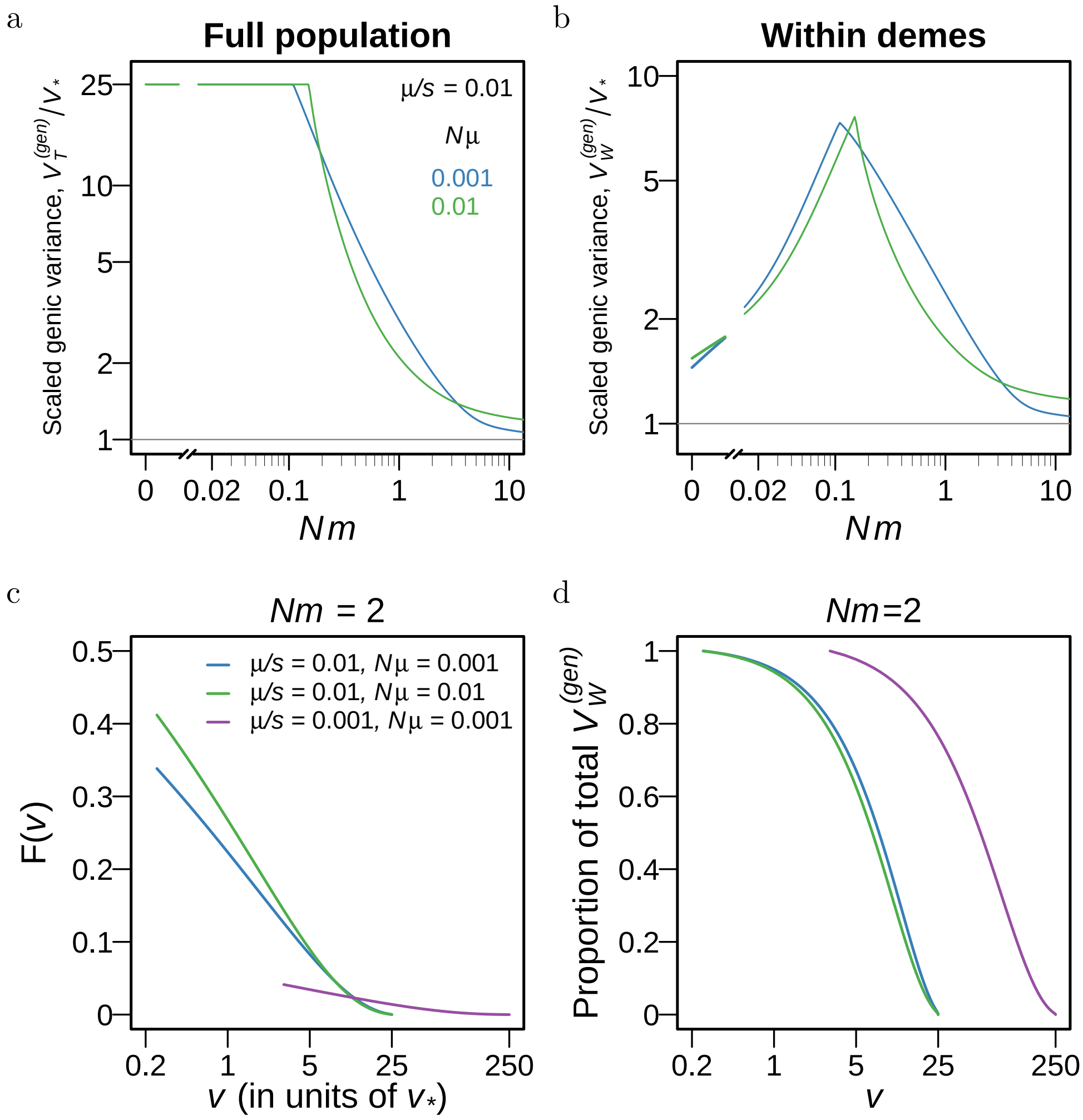

Figure S7. (a),(b): The expected (a) total genic variance across the full population, $V_T^{(gen)}/V_*$, and (b) within-deme genic variance, $V_W^{(gen)}/V_*$, as a function of Nm, for a trait influenced by $L = 200$ equal-effect loci. Genic variances are scaled by $V_* = 4\mu L V_s$. The different colors show theoretical LD predictions for different values of Ns and $N\mu$ (with $\mu/s = 0.01$ held fixed).
(c),(d): (c) Cumulative probability $F(v)$ that the variance contributed by a locus exceeds v, and (d) proportion of within-deme genic variance $V_W^{(gen)}$ explained by loci with variance exceeding v, as a function of v, for a trait influenced by $L = 200$ loci of equal effect, for $Nm = 2$. All per-locus variances are measured in units of $v_* = 4\mu V_s$. The different colors show theoretical LD predictions for different values of $N\mu$, keeping either μ/s or Ns fixed.

References

Barton, N. H. and Rouhani, S. (1993). Adaptation and the 'shifting balance'. *Genetics Research*, 61(1):57–74.

Kobayashi, Y., Hammerstein, P., and Telschow, A. (2008). The neutral effective migration rate in a mainland-island context. *Theoretical Population Biology*, 74(1):84–92.

Sachdeva, H. (2022). Reproductive isolation via polygenic local adaptation in sub-divided populations: Effect of linkage disequilibria and drift. *PLOS Genetics*, 18(9):e1010297.

Surendranadh, P. and Sachdeva, H. (2025). Effect of assortative mating and sexual selection on polygenic barriers to gene flow. *Evolution*, 79(7):1185–1198.

Veller, C. and Simons, Y. (2024). Stabilizing selection generates selection against introgressed dna. *bioRxiv*.

Westram, A. M., Stankowski, S., Surendranadh, P., and Barton, N. (2022). What is reproductive isolation? *Journal of Evolutionary Biology*, 35(9):1143–1164.
